# Disruption of alpha-tubulin releases carbon catabolite repression and enhances enzyme production in *Trichoderma reesei* even in the presence of glucose

**DOI:** 10.1186/s13068-021-01887-0

**Published:** 2021-02-08

**Authors:** Nozomu Shibata, Hiroshi Kakeshita, Kazuaki Igarashi, Yasushi Takimura, Yosuke Shida, Wataru Ogasawara, Tohru Koda, Tomohisa Hasunuma, Akihiko Kondo

**Affiliations:** 1 Biological Science Research, Kao Corporation, 1334 Minato, Wakayama, Wakayama 640-8580 Japan; 2 Graduate School of Science, Technology and Innovation, Kobe University, 1-1 Rokkodai, Nada-ku, Kobe, Hyogo 657-8501 Japan; 3 Biological Science Research, Kao Corporation, 2606 Akabane, Ichikai, Haga, Tochigi, 321-3497 Japan; 4 Department of Bioengineering, Nagaoka University of Technology, 1603-1 Kamitomioka, Nagaoka, Niigata, 940-2188 Japan; 5 Engineering Biology Research Center, Kobe University, 1-1 Rokkodai, Nada-ku, Kobe, Hyogo 657-8501 Japan; 6 Department of Chemical Science and Engineering, Graduate School of Engineering, Kobe University, 1-1 Rokkodai, Nada-ku, Kobe, 657-8501 Japan

**Keywords:** *Trichoderma reesei*, Biomass saccharification enzyme, Cellulase, Hemicellulase, Carbon catabolite repression, Glucose resistant, Alpha-tubulin

## Abstract

**Background:**

*Trichoderma reesei* is a filamentous fungus that is important as an industrial producer of cellulases and hemicellulases due to its high secretion of these enzymes and outstanding performance in industrial fermenters. However, the reduction of enzyme production caused by carbon catabolite repression (CCR) has long been a problem. Disruption of a typical transcriptional regulator, Cre1, does not sufficiently suppress this reduction in the presence of glucose.

**Results:**

We found that deletion of an α-tubulin (*tubB*) in *T. reesei* enhanced both the amount and rate of secretory protein production. Also, the tubulin-disrupted (Δ*tubB)* strain had high enzyme production and the same enzyme profile even if the strain was cultured in a glucose-containing medium. From transcriptome analysis, the Δ*tubB* strain exhibited upregulation of both cellulase and hemicellulase genes including some that were not originally induced by cellulose. Moreover, cellobiose transporter genes and the other sugar transporter genes were highly upregulated, and simultaneous uptake of glucose and cellobiose was also observed in the Δ*tubB* strain. These results suggested that the Δ*tubB* strain was released from CCR.

**Conclusion:**

*Trichoderma reesei* α-tubulin is involved in the transcription of cellulase and hemicellulase genes, as well as in CCR. This is the first report of overcoming CCR by disrupting α-tubulin gene in *T. reesei*. The disruption of α-tubulin is a promising approach for creating next-generation enzyme-producing strains of *T. reesei.*

## Background

Cellulose and hemicellulose are naturally produced in higher plants as cell wall components from CO_2_ and are the two most abundant polysaccharides on Earth. Using this biomass is important to break the social structure of mass consumption and mass disposal that depends on petrochemicals, allowing us to build a sustainable society. Hydrolysis of these polysaccharides with enzymes such as cellulase and hemicellulase provides sugars that can be converted by microbial fermentation into biofuels, bio-based commodity chemicals, and value-added products [1]. *Trichoderma reesei,* an anamorph of *Hypocrea jecorina,* is a very important filamentous fungus used to produce industrial cellulases and hemicellulases. The cellulase market is projected to reach about 0.8 billion USD in 2025 [2], and there have been many studies on enzyme production by *T. reesei* [3–6].

Enzyme production is a major focus of research and development. Cellulosic biomass has a strong and complex structure, with hemicellulose and lignin surrounding crystalline cellulose [7]. It is therefore necessary to increase cellulase production and improve its enzymatic hydrolysis efficiency [8].

The saccharification of cellulosic biomass requires the synergistic effects of various enzymes, such as cellobiohydrolases (CBH, EC 3.2.1.91), which release cellobiose from the ends of crystalline cellulose, endo-β-1,4-glucanases (EG, EC 3.2.1.4), which cleave internal bonds of amorphous cellulose, and β-glucosidases (BGL, EC 3.2.1.21), which convert cellobiose and cello-oligosaccharides into glucose to prevent product inhibition of CBH by cellobiose. These enzymes are collectively referred to as cellulases. Hemicellullose is also synergistically degraded by endo-β-1,4-xylanases (XYN, EC 3.2.1.8), β-xylosidases (BXL, 3.2.1.37), and other accessory enzymes. These enzymes involved in the degradation of hemicellulose are collectively referred to as hemicellulases.

The well-studied fungus *Trichoderma reesei* produces a complete set of cellulases in abundance when cultivated with inducer such as cellulose, cellobiose and sophorose as reviewed in [9, 10]. The CBH and EG activities of *T. reesei* are higher than those of other microorganisms, but its BGL activity is lower than that of cellulase mixes from other organisms [11] and results in cellobiose accumulation and subsequent product inhibition of CBH, reducing enzymatic hydrolysis efficiency [12]. This problem has been addressed by characterizing the BGLs and overexpressing the BGL genes in *T. reesei* [13–15]. In a previous study, we conducted the heterologous production of AaBGL1 (BGL1 from *Aspergillus* *aculeatus*) by *T. reesei* to enhance the degradation of NaOH-pretreated rice straw, thereby reducing the amount of cellulase required [15, 16].

The cellulase and hemicellulase genes in *T. reesei* are regulated by transcriptional activators (Xyr1, Ace2 and Ace3) and repressors (Ace1 and Cre1) [17–21]. Cre1 is a regulator of CCR. When *T. reesei* is cultivated in medium containing glucose, Cre1 represses cellulase and hemicellulase gene expression to prioritize the uptake of glucose, resulting in little cellulase production. The hyper-secreting *T. reesei* mutant RUT-C30 contains a *cre1* gene truncated by mutagenesis and has higher cellulase and hemicellulase production in glucose-containing media than its parent strain QM6a [22, 23]. Similarly, the strain PC-3-7, obtained as a mutant capable of producing cellulase on agar plates containing glucose, has a mutation in *cre1* [24, 25]. Although cellulase genes such as *cbh1* are expressed in the presence of glucose in this *cre1*-deficient strain [26], cellulase production is sharply decreased in a glucose concentration-dependent manner [27], suggesting that even if *cre1* is knocked out, enzyme production is repressed under high glucose concentration. Continuous but low glucose feed levels in fed-batch cultures using sugar solutions containing glucose prevent the repression of enzyme production by glucose [28, 29]. However, this approach requires accurate control of the sugar flow rate to avoid glucose accumulation and thus new strains resistant to CCR are desirable.

Tubulin is a protein that forms microtubules and centrosomes in eukaryotic cells. There are two types of tubulin, alpha-tubulin and beta-tubulin, and these heterodimers polymerize to form the protofilaments of microtubules. Microtubules are formed when these protofilaments are joined into tubular structures and function in many essential cellular processes such as mitosis [30]. Fungal alpha- and beta-tubulins are classified as α_1_, α_2_, α_3_, β_1_ and β_2_ by phylogenetic analysis [31]. α_1_-Tubulins are found in all fungi examined to date, but α_2_-tubulins are found only in Ascomycota (except for Saccharomycetes and Taphrinomycotina) and α_3_-tubulins are found only in some Basidiomycetes. β_1_-Tubulins are found in all fungi but β_2_-tubulins are found only in Basidiomycetes and *Yarrowia lipolytica*. The two alpha-tubulins of *A. nidulans*, TubA (NCBI Reference Sequence: XP_657920.1) and TubB (NCBI Reference Sequence: XP_680839.1), belong to the α_1_ and α_2_-tubulin classes, respectively, and their functions were previously analyzed [32]. TubA is involved in nuclear division and TubB may be involved in germination. Deletion of the *tubB* gene of *A. nidulans* had no detectable effect on vegetative growth or asexual reproduction but the *tubB* gene is essential for sexual development [33]. In addition, genomic analysis of *A. nidulans* revealed two other α_2_-tubulin gene copies, and these tubulins likely have distinct functions. Genomic analysis of *T. reesei* showed that it has one α_1_-tubulin, one α_2_-tubulin, and two β_1_-tubulins.

In this study, we evaluated the protein production of the three recombinant strains of *T. reesei* PC-3-7 expressing BGL from *A. aculeatus*, which has been constructed in previous studies, and found that the protein production was decreased in two strains. But one strain, E1AB1, led to an unexpectedly phenotype of improvement of secretory protein. We hence conducted in-depth analysis of this strain and found that gene encoding alpha-tubulin was disrupted.

Sugar consumption during mixed-substrate fermentation and transcriptome analysis of the tubulin-disrupted strain clearly indicate a release from CCR. This is the first report of overcoming CCR by disrupting the tubulin-encoding gene to increase cellulase production in *T. reesei*.

## Results

### Protein production in *aabgl1*-expressing recombinants

**Fig. 1:**
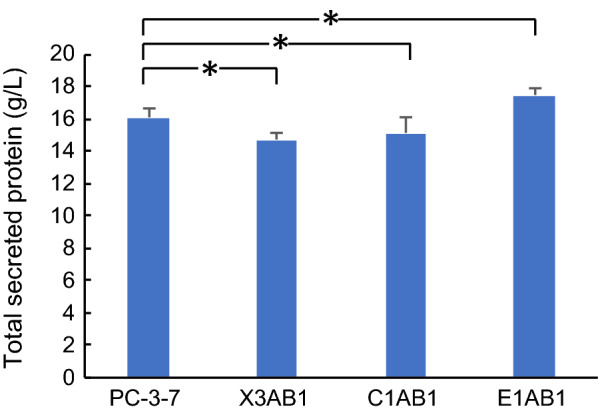
Secretory protein production by the *aabgl1* recombinant strains and parent strain PC-3-7. Fermentation was conducted using 10% (w/v) microcrystalline cellulose and 2% (w/v) beechwood xylan as carbon sources. Data are expressed as mean ± SD of three biological replicates. Statistical significance was determined by two-tailed unpaired Student’s t-test. *p < 0.05

We investigated the effect of overexpression of BGL on cellulase production in *T. reesei* by culturing the recombinant strains X3AB1, C1AB1 and E1AB1 expressing *A. aculeatus bgl1* (*aabgl1*) under the control of the *xyn3*, *cbh1* and *egl1* promoters, respectively (Table 1). In the X3AB1 strain, the *aabgl1* expression cassette was integrated into the *xyn3* locus homologously, while the C1AB1 and E1AB1 strains were constructed by non-homologous insertion of the *aabgl1* cassette. The strains were cultured in 2-L jar fermenters using 10% (w/v) cellulose and 2% (w/v) xylan as carbon sources. Protein production stopped when the ammonia solution, which is a nitrogen source and is used to keep the pH constant, was no longer supplied. So, the endpoint of the cultivation was the sampling point after the ammonia solution was no longer supplied. As shown in Fig. 1, X1AB1 and C1AB1 produced 14.7 g/L and 15.1 g/L protein, respectively, which are lower than the protein production levels of PC-3-7 (16.0 g/L, *p* < 0.05). E1AB1 produced 17.4 g/L protein, which is significantly higher than PC-3-7 (*p* < 0.05).

**Table 1:** *T. reesei* strains used and generated within this study

Strains	Description or genotypes	Reference
PC-3-7 (ATCC 65589)	Hyper-secreting mutant	20
X3AB1	*amdS*^+^, Δ*xyn3*:: *xyn3*p-*aabgl1*	11
C1AB1	*amdS*^+^, *cbh1*p-*aabgl1*	12
E1AB1	*amdS*^+^, *egl1*p-*aabgl1*	12
PC-3-7Δ*tubB*	*amdS*^+^,Δ*tubB*	This study
PC-3-7Δ*tubB::Pegl1-aabgl1*	*amdS*^+^,Δ*tubB::egl1*p-*aabgl1*	This study

**Table 2:** Morphological features of PC-3-7 and PC-3-7Δ*tubB* grown in glucose medium and in cellulose medium

	Glucose medium		Cellulose medium	
	PC-3-7	PC-3-7Δ*tubB*	PC-3-7	PC-3-7Δ*tubB*
Cell length (μm)	12.0 ± 5.3*	16.9 ± 8.4*	11.4 ± 4.0	11.2 ± 5.7
Cell diameter (μm)	2.5 ± 0.5	2.3 ± 0.4	2.1 ± 0.4*	3.2 ± 1.0*
Cell ratio (length/diameter)	4.9 ± 2.2*	7.7 ± 4.1*	5.6 ± 2.1*	4.0 ± 2.7*

^*^Significant at *p * <  0.01 between the strains in the same medium condition

### Identification of the expression cassette integration site in E1AB1

We determined the reason for increased protein production in E1AB1 by identifying the locus of the gene insertion in E1AB1 using inverse PCR. As shown in Additional file 1: Fig. S1a, the *aabgl1*-expressing cassette was inserted in the middle of the DNA encoding alpha-tubulin B (gene ID: 120830, hereinafter called *tubB*). PCR amplification from the start codon to the stop codon of *tubB* showed that the full length of the open reading frame (ORF) was amplified in PC-3-7, whereas a PCR fragment of *tubB* ORF with inserted *aabgl1*-expressing cassette was amplified in E1AB1 (Additional file 1: Fig. S1b). In addition, PCR amplification from *amdS* in the *aabgl1*-expressing cassette to upstream of *tubB* in the genome was observed only in E1AB1. These results indicated that the *aabgl1*-expressing cassette was inserted in *tubB* locus. Thus, higher protein production by E1AB1 may be related to the disruption of *tubB*. Alpha-tubulin polymerizes with beta-tubulin into microtubules and genome analysis has shown that there are two alpha-tubulin genes (gene ID: 120789 and 120830) in *T. reesei* [31]. Although microtubules are a major component of the eukaryotic cytoskeleton, and microtubules function in many essential cellular processes such as mitosis [30], no decrease in growth rate or glucose consumption was observed in E1AB1 (Additional file 2: Fig. S2).

### Construction of a *tubB*-deleted strain

To examine the effect of disruption of *tubB* gene, we constructed *tubB*-deleted strains. In one strain, PC-3-7 was transformed with linearized pUC-Δ*tubB*-*amdS*, in which the *tubB* ORF was replaced by the *amdS* marker. In the other strain, PC-3-7 was transformed with pUC-Δ*tubB*-*Pegl1-aabgl1-amdS* containing the 5′ and 3′ flanking regions of the *tubB* ORF linked with the *aabgl1* ORF under the control of the *egl1* promoter and *amdS* marker. In each case, a homologous recombination transformant was identified by colony PCR analysis, providing the transformant of pUC-Δ*tubB*-*amdS*, designated PC-3-7Δ*tubB,* and the transformant of pUC-Δ*tubB*-*Pegl1-aabgl1-amdS*, designated PC-3-7Δ*tubB::Pegl1-aabgl1*.

**Fig. 2:**
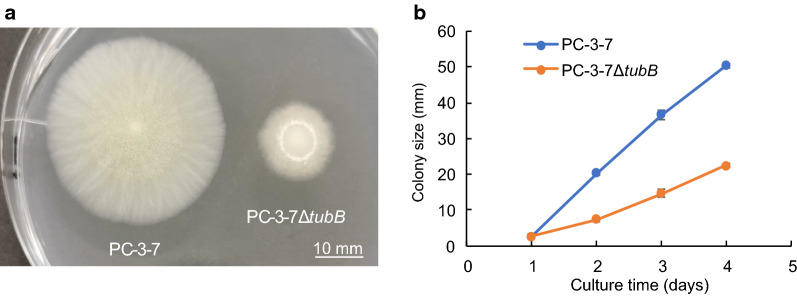
Colony morphology of PC-3-7 and PC-3-7Δ*tubB.* **a** Colonies of PC-3-7 and PC-3-7*ΔtubB* formed on potato dextrose agar (PDA) plate at 30 °C for 3 days. Scale bar indicates 10 mm. **b** Colony size of PC-3-7 and PC-3-7*ΔtubB* incubated on PDA plate. Data are expressed as mean ± SD of three biological replicates

To observe the effect of *tubB* disruption on morphology, a growth assay of PC-3-7Δ*tubB* and PC-3-7 on PDA plates was performed. The mycelial morphology on PDA plate is shown in Fig. 2a and the change in colony size over time is shown in Fig. 2b. The growth speed of PC-3-7Δ*tubB* on PDA plate was obviously lower than that of PC-3-7. However, this decrease of growth rate was not observed when PC-3-7Δ*tubB* was cultured in liquid medium (Additional file 2: Fig. S2).

**Fig. 3:**
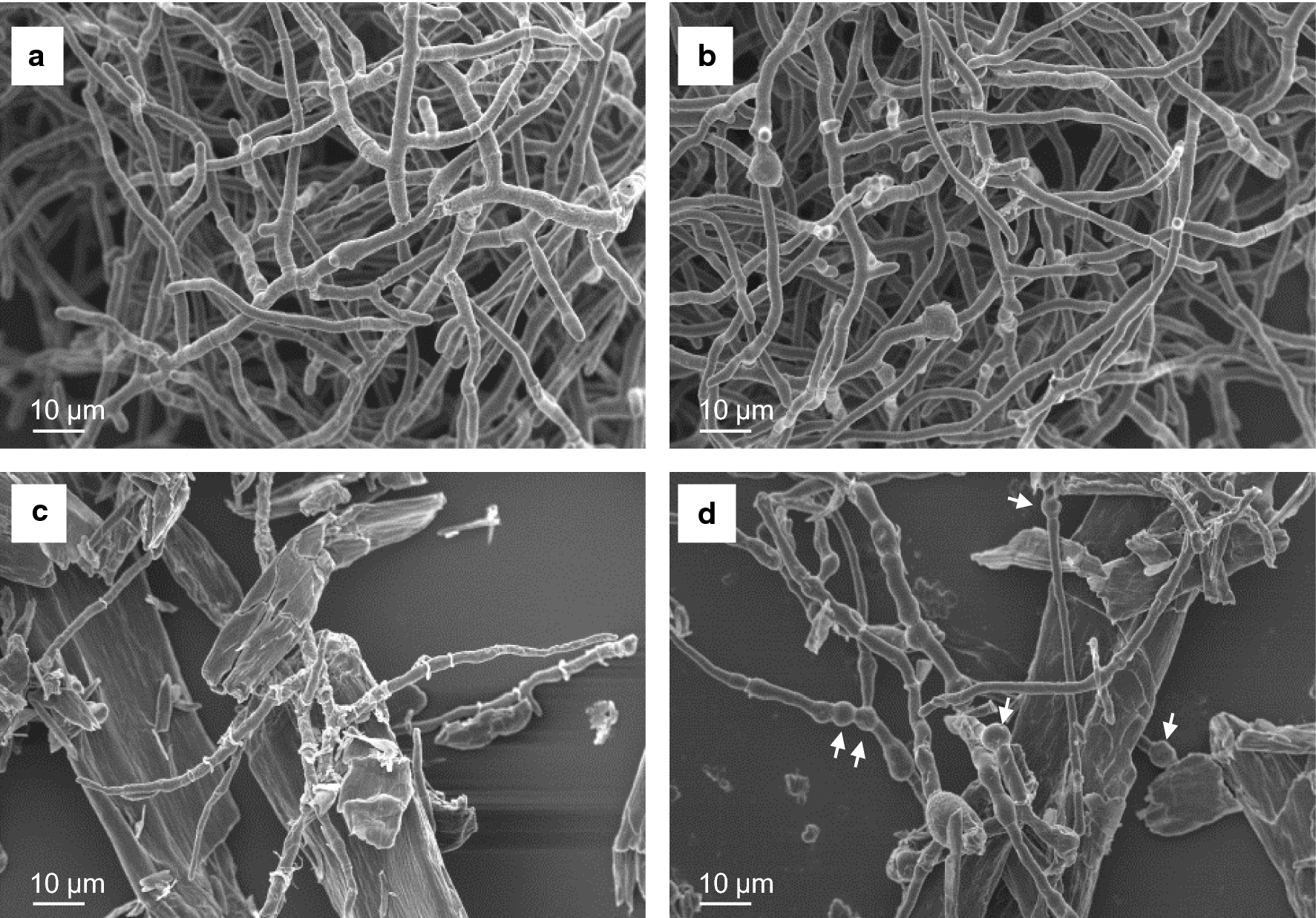
SEM images of PC-3-7 and PC-3-7Δ*tubB.* SEM images of *T. reesei* PC-3-7 (**a** and **c**) and PC-3-7Δ*tubB* (**b** and **d**) mycelia grown in liquid culture containing 1% (w/v) glucose for 1 day (**a**, **b**) and 1% (w/v) cellulose for 3 days (**c**,** d**). Scale bar indicates 10 µm. White arrows indicate the rounded hypha

The hyphae of PC-3-7Δ*tubB* cultured in liquid medium containing glucose or cellulose were compared with PC-3-7 using scanning electron microscopy (SEM) (Fig. 3). There was no difference in the SEM images of these strains when cultivated in glucose medium (Fig. 3a, b). However, when cultivated in cellulose medium, rounded hyphae were frequently observed in the SEM image of PC-3-7Δ*tubB* (Fig. 3c, d). The mean cell length and diameter values for 70 cells randomly selected from each SEM image are shown in Table 2. PC-3-7Δ*tubB* cells cultivated in glucose were longer than PC-3-7 cells under the same conditions. When the cells were cultivated with cellulose, the cell diameters of PC-3-7Δ*tubB* were significantly larger than those of PC-3-7. The cell ratio (length/diameter) of PC-3-7Δ*tubB* was significantly larger than that of PC-3-7 when cultured with glucose and significantly smaller when cultured with cellulose. These results indicate that PC-3-7Δ*tubB* cells are more elongated when cultured with glucose and more rounded when cultured with cellulose, suggesting that the disruption of *tubB* affects cell morphology cultured in liquid medium.

### Evaluation of secretory protein production of *tubB*-deleted strains

**Fig. 4:**
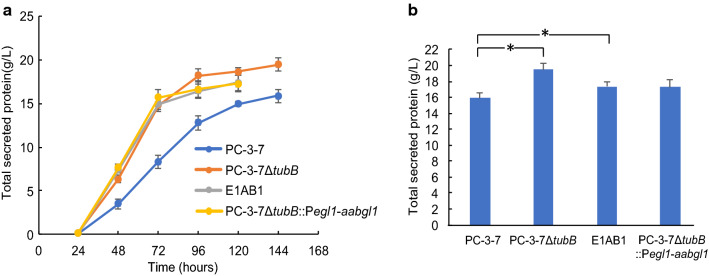
Secretory protein production by the *tubB*-deficient strains and parent strain PC-3-7. **a** Time course of the amount of secretory protein. Fermentation was conducted using 10% (w/v) microcrystalline cellulose and 2% (w/v) beechwood xylan as carbon sources. Data are expressed as mean ± SD of three biological replicates. **b** Amount of secreted protein at the end of cultivation. Data are expressed as mean ± SD of three biological replicates. Statistical significance was determined by two-tailed unpaired Student’s t-test. *p < 0.05

We compared the amount of secretory protein produced by the constructed *tubB*-deleted strains by cultivating in 2-L jar fermenters using 10% (w/v) cellulose and 2% (w/v) xylan as carbon sources (Fig. 4). The endpoint of the cultivation was the sampling point after the ammonia solution, which is a nitrogen source and used to keep the pH constant, was no longer supplied, and the changes in the amount of secreted protein are shown in Fig. 4a, and the amount of secreted protein at the endpoint is shown in Fig. 4b. The PC-3-7Δ*tubB* and E1AB1 showed significantly higher secretory protein production (17.4—19.5 g/L) than the parent PC-3-7 (16.0 g/L), with PC-3-7Δ*tubB* showing the highest secretory protein production. The cultivations of E1AB1 and PC-3-7Δ*tubB::Pegl1-aabgl1* were complete in 120 h while PC-3-7 and PC-3-7Δ*tubB* required 144 h. Initial protein production rates by the Δ*tubB* strains were especially higher than that of PC-3-7 (Fig. 4a). These results indicate that disruption of the *tubB* gene enhances both the amount and rate of protein secretion.

### Effects of glucose on protein production by *tubB*-deficient strains

**Fig. 5:**
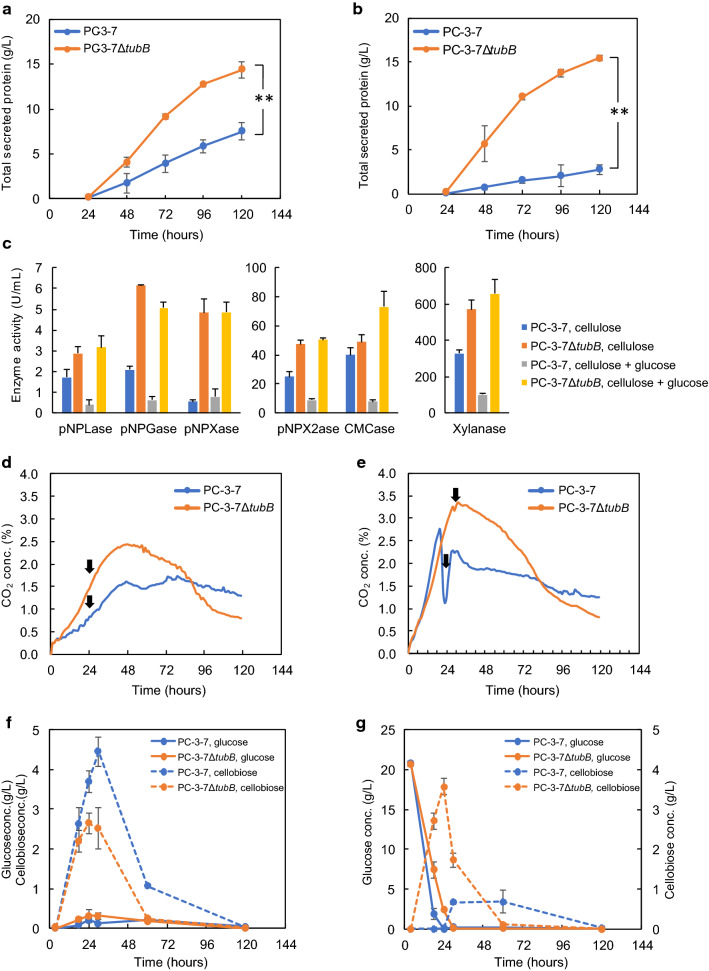
Effects of glucose on protein production, respiration, and sugar uptake of PC-3-7 and PC-3-7Δ*tubB*. **a** Secretory protein production, **d** the concentration of CO_2_ in the exhaust gas and **f** glucose and cellobiose concentration when cultivated in medium with 10% (w/v) microcrystalline cellulose. **b** Secretory protein production, **e** the concentration of CO_2_ in the exhaust gas and **g** glucose and cellobiose concentration when cultivated in medium with 10% (w/v) microcrystalline cellulose and 2.5% (w/v) glucose. Blue lines indicate PC-3-7 and orange lines indicate PC-3-7Δ*tubB*. **c** Cellulolytic and hemicellulolytic enzyme activities in the culture supernatant after 120 h of cultivation, including pNPGase (the BGL activity), pNPLase (the CBH1 activity), CMCase (the EG activity), pNPXase (the BXL activity), pNPX2ase and xylanase (the XYN activity). In **f** and **g**, solid lines indicate glucose and dashed lines indicate cellobiose. Data are expressed as mean ± SD of three biological replicates. For secretory protein production, statistical significance was determined by two-tailed unpaired Student’s t-test. **p < 0.01. The arrows in **c** and **e** indicate the time at which cells were collected for RNA-seq analysis

Since high expression of BGL improves the release of glucose from cellulose, we investigated the effect of glucose on *tubB*-deficient strains by culturing PC-3-7Δ*tubB* and PC-3-7 in 2-L jar fermenters using 10% (w/v) cellulose with and without 2.5% (w/v) glucose for 120 h. When cultured using only cellulose (Fig. 5a), PC-3-7Δ*tubB* showed higher protein secretion (14.4 g/L) than PC-3-7 (7.5 g/L). When cultured using cellulose and glucose (Fig. 5b), protein secretion by PC-3-7 was extremely low (2.8 g/L) whereas PC-3-7Δ*tubB* produced more protein (15.4 g/L) than when cultured with only cellulose. To confirm whether the amount of cellulase and hemicellulase increased along with the increase in the amount of secreted protein, the cellulase (pNPGase, pNPLase, and CMCase) and hemicellulase (pNPXase, pNPX2ase, and xylanase) activities in the culture supernatant were measured after 120 h of cultivation. The results of enzymatic assay are shown in Fig. 5c, and specific activity and the SDS-PAGE of the culture broth are shown in Additional file 3: Fig. S3. Each enzyme activity (Fig. 5c) was consistent with the amount of secreted protein (Fig. 5a, b), suggesting that enzyme production was enhanced in PC-3-7Δ*tubB* compared to PC-3-7. There was no difference in each specific activity, except for the pNPXase activity of PC-3-7 when cultured with only cellulose (Additional file 3: Fig. S3a). SDS-PAGE of PC-3-7Δ*tubB* showed no difference in protein composition and amount with and without glucose (Additional file 3: Fig. S3b, c). In contrast, PC-3-7 exhibited decreased amount of secretory protein and production of an unknown protein of about 75 kDa following the addition of glucose.

Figure 5d, e shows the concentration of the exhaust CO_2_ during fermentation. As shown in Fig. 5d, PC-3-7Δ*tubB* generated more CO_2_ by respiration than PC-3-7 even at the early stage of culture, indicating that PC-3-7Δ*tubB* can immediately utilize cellulose as a carbon source. When cultured in medium containing both cellulose and glucose, PC-3-7 generated more CO_2_ than PC-3-7Δ*tubB* up to 20 h, then a sharp drop was observed at 20–23 h. PC-3-7Δ*tubB* cultured in the same medium exhibited a slight reduction in CO_2_ generation at 28 h (Fig. 5e).

Figure 5f, g shows the glucose and cellobiose concentration in the culture broth. The cultivation of PC-3-7 and PC-3-7Δ*tubB* in medium containing only cellulose (Fig. 5f) resulted in barely detectable levels of glucose whereas cellobiose was detectable in the culture broth. Cellobiose accumulated in the medium between 24 and 30 h, and PC-3-7 accumulated more cellobiose than PC-3-7Δ*tubB*. When cultivated in medium containing both cellulose and glucose (Fig. 5g), PC-3-7 showed a higher glucose consumption rate than PC-3-7Δ*tubB*, and the glucose was depleted between 18 and 24 h. The sharp drop in exhaust CO_2_ was likely caused by switching the assimilating carbon source from glucose to cellulose (Fig. 5e).

### RNA-seq analysis of the *tubB*-deficient strain PC-3-7Δ*tubB*

PC-3-7Δ*tubB* shows high protein production even in the presence of glucose. We investigated the underlying mechanism caused by the deletion of *tubB* by determining the transcription profile of PC-3-7Δ*tubB* by RNA-sequencing (RNA-seq) analysis. PC-3-7 and PC-3-7Δ*tubB* were cultured in 2-L jar fermenters using 10% (w/v) cellulose with and without 2.5% (w/v) glucose. Cells cultured in medium with only cellulose were collected after 24 h (PC-3-7_24h_C and Δ*tubB*_24h_C) and 48 h (PC-3-7_48h_C and Δ*tubB*_48h_C). Cells cultivated in medium containing both cellulose and glucose were collected after the CO_2_ concentration decreased to adjust the growth phase (24 h for PC-3-7; PC-3-7_24h_C + G, 30 h for PC-3-7Δ*tubB*; Δ*tubB*_30h_C + G), and 48 h (PC-3-7_48h_C + G and Δ*tubB*_48h_C + G). RNA-seq was performed on the extracted RNA samples and normalized gene expression (RPKM, reads per kilobase per million) was calculated for all 9,115 coding sequences.

The RPKM value of the *tubB* gene was 0 in all PC-3-7Δ*tubB* samples, clearly indicating that *tubB* was deleted in PC-3-7Δ*tubB*. The RPKM values of the other tubulin genes were unaffected by deletion of the *tubB* gene (Additional file 4: Table S1).

**Fig. 6:**
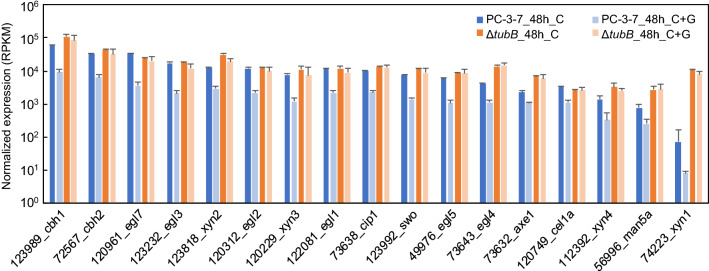
Normalized expression of major CAZymes in PC-3-7 and PC-3-7Δ*tubB.* Blue bars indicate PC-3-7 and orange bars indicate PC-3-7Δ*tubB*. C, cultivated with cellulose; C + G, cultivated with cellulose and glucose. Data are expressed as mean ± SD of three biological replicates

The highest expression levels of carbohydrate-active enzymes (CAZymes) were obtained for *cbh1*, *cbh2*, *egl1*, *egl2*, *egl3*, *egl4*, *egl5*, *xyn2*, *cip1* and *swo*, with *cbh1* being the highest expressed gene under all conditions except PC-3-7_48h_C + G (Fig. 6 and Additional file 5: Table S2). Interestingly, *xyn1* was highly expressed by PC-3-7Δ*tubB* under all conditions but at extremely low levels in PC-3-7. The gene expression of transcription factors of cellulases and hemicellulases is listed in Additional file 6: Table S3; *ace1*, *vel1* and *vib1* were highly expressed in PC-3-7, and the expression of *cre1* was slightly elevated in PC-3-7Δ*tubB*.

### DEG analysis between PC-3-7 and PC-3-7Δ*tubB*

**Fig. 7:**
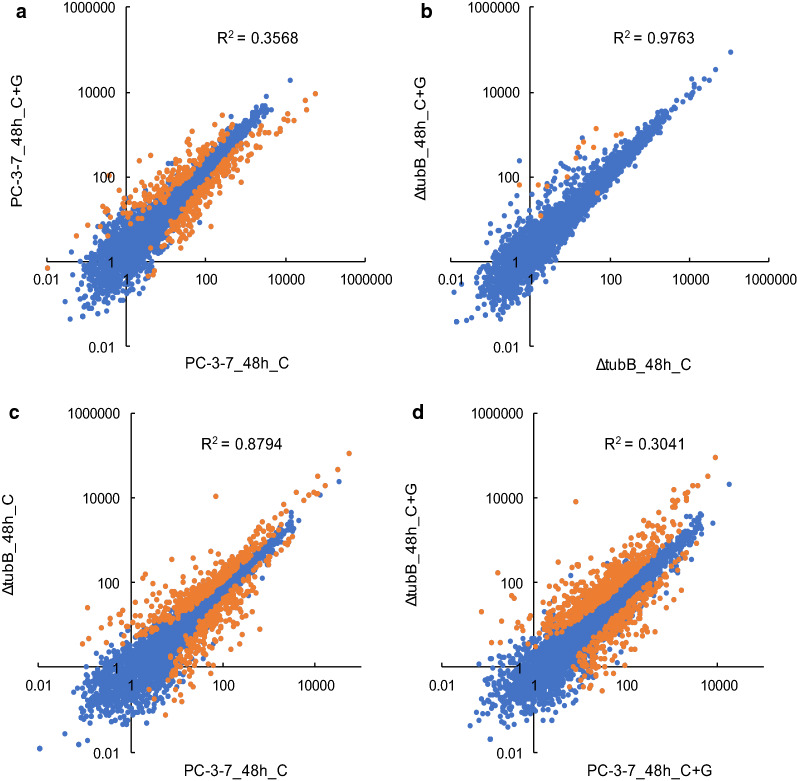
Scatter plots of gene expression profiles. Scatter plots between PC-3-7_48h_C and PC-3-7_48h_C + G (**a**) and Δ*tubB*_48h_C, ΔtubB_48h_C + G (**b**), PC-3-7_48h_C and Δ*tubB*_48h_C, and PC-3-7_48h_C + G and Δ*tubB*_48h_C + G. The x- and y-axis represent RPKM value of all CDSs. The DEGs identified in DEseq analysis are highlighted in orange color

We analyzed the transcriptional differences by differentially expressed gene (DEG) analysis using the R and DESeq2 package. We identified 416 DEGs (187 upregulated and 229 downregulated) between PC-3-7_48h_C and PC-3-7_48h_C + G, 13 DEGs (12 upregulated and 1 downregulated) between Δ*tubB*_48h_C and Δ*tubB*_48h_C + G, 744 DEGs (478 upregulated and 266 downregulated) between PC-3-7_48h_C and Δ*tubB*_48h_C, and 803 DEGs (545 upregulated and 258 downregulated) between PC-3-7_48h_C + G and Δ*tubB*_48h_C + G with adjusted *p* < 0.01. Scatter plots of gene expression profiles compared above are shown in Fig. 7 and those that were identified to be DEGs were highlighted. Moreover, correlation analyses of the RPKM values of all coding sequences (CDSs) are shown in Additional file 7: Table S4. There was a high correlation (R = 0.988, R^2^ = 0.976) between Δ*tubB*_48h_C and Δ*tubB*_48h_C + G (Fig. 7b) but no correlation (R = 0.597, R^2^ = 0.357) between PC-3-7_48h_C and PC-3-7_48h_C + G (Fig. 7a). In both strains, cellulase genes are induced when cellulose is the sole carbon source. The addition of glucose totally altered gene expression in PC-3-7 but had almost no effect on PC-3-7Δ*tubB* cellulase gene expression, showing that cellulase gene expression in PC-3-7Δ*tubB* was induced even in the presence of glucose.

The 744 DEGs between PC-3-7_48h_C and Δ*tubB*_48h_C contained 74 CAZymes, 57 of which were highly expressed in PC-3-7Δ*tubB* (Fig. 7c), and the 803 DEGs between PC-3-7_48h_C + G and Δ*tubB*_48h_C + G contained 75 CAZymes, 63 of which were highly expressed in PC-3-7Δ*tubB* (Fig. 7d). Cellulase genes such as *cbh1, 2, egl4, 5* and *6* were particularly significantly expressed in PC-3-7Δ*tubB* in both C and C + G condition (Additional file 8: Table S5). Moreover, other CAZymes, such as alpha-glucuronidase (gene ID: 72526; *aguA*), alpha-D-galactosidase (72704), acetyl xylan esterases (54219 and 73632; *axe1*), xylanases (*xyn1, 2, 3, 4* and *5*) and pectinase (103049) were significantly expressed in PC-3-7Δ*tubB* in both C and C + G condition. Knockout of *tubB* thus increased the expression of CAZymes not originally induced by cellulose.

### Gene ontology enrichment analysis

We analyzed the effect of deletion of the *tubB* gene by gene ontology (GO) enrichment analysis of the DEGs between PC-3-7_48h_C and Δ*tubB*_48h_C. GO enrichment analysis was performed using the R and GOSeq package. For each enriched GOs, the number of genes, the number of DEGs, and the number of DEGs that are upregulated in PC-3-7Δ*tubB* were listed in Additional file 9: Table S6. The most enriched GOs were GO:0005975 (carbohydrate metabolic process) and GO:0004553 (hydrolase activity, hydrolyzing O-glycosyl compounds) and most of the DEGs corresponding to these GOs were highly expressed in PC-3-7Δ*tubB*. Since the genes corresponding to these GOs are glycoside hydrolase, the most significant effect of the knockout of *tubB* was the change in the expression of CAZymes. In addition, GOs related to transporter such as GO:0006810 (transporter), GO:0016020 (membrane), GO:0006865 (amino acid transport), GO:0005351 (carbohydrate:proton symporter activity) and GO:0008643 (carbohydrate transport) were identified in the enriched GOs and where the corresponding DEGs were highly expressed in PC-3-7Δ*tubB*, indicating that the expression levels of many transporters were simultaneously upregulated due to deletion of the *tubB* gene. Twenty-three sugar transporter-related genes (GO:0005351 or GO:0008643, or annotated genes encoding sugar transporters) were differentially expressed between PC-3-7_48h_C and Δ*tubB*_48h_C and 19 genes were upregulated in PC-3-7Δ*tubB*. The most significantly upregulated gene was gene ID 69957 or *Tr69957* (log2 fold change = 6.15), reported as a cellobiose, mannose and xylose transporter [34]. ID 3405 or *crt1*, reported as a cellobiose and sophorose transporter which plays a key role in the cellulolytic signaling process [35, 36], showed the highest gene expression level of the significantly expressed transporters. ID 50894 or *str1*, reported as a transporter induced by pentose, ID 121482 or *str2*, reported as a transporter with broad substrate specificity [37], and ID 79202 and 77517, reported as a lactose transporter [38], were all upregulated in PC-3-7Δ*tubB*.

### Analysis of sugar transport

**Fig. 8:**
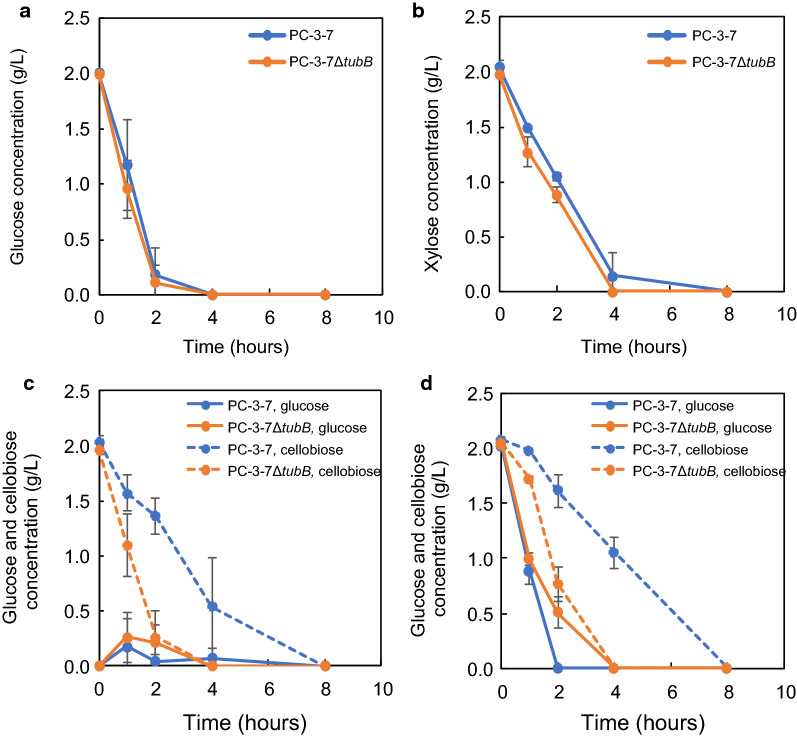
Sugar consumption by PC-3-7 and PC-3-7Δ*tubB* Blue lines indicate PC-3-7 and orange lines indicate PC-3-7Δ*tubB*. **a** Glucose concentration after 0.2% (w/v) glucose was added. **b** Xylose concentration after 0.2% (w/v) xylose was added. **c** Glucose and cellobiose concentrations in the supernatant after 0.2% (w/v) cellobiose was added. Solid lines indicate glucose concentration and dashed lines indicate cellobiose concentration. **d** Glucose and cellobiose concentrations in the supernatant after 0.2% (w/v) glucose and 0.2% (w/v) cellobiose were added. Solid lines indicate glucose concentration and dashed lines indicate cellobiose concentration. Data are expressed as mean ± SD of three biological replicates

Based on these RNA-seq analyses, knockout of *tubB* may alter sugar transport ability and thus we analyzed the uptake rates of glucose, xylose, cellobiose, and a mixture of glucose and cellobiose in PC-3-7 and PC-3-7Δ*tubB*. The cells were cultured in 1% (w/v) glucose medium for 2 days, then each sugar was added at a final concentration of 0.2% (w/v) and the sugar concentration in the medium was monitored (Fig. 8). There was no difference in sugar uptake between PC-3-7 and PC-3-7Δ*tubB* when glucose (Fig. 8a) or xylose (Fig. 8b) was added. In both strains, glucose was depleted in about 2 h and xylose was consumed in about 4 h. When cellobiose was added, PC-3-7Δ*tubB* showed a higher consumption rate than PC-3-7. PC-3-7Δ*tubB* assimilated 0.2% (w/v) cellobiose in about 2 h while PC-3-7 could not consume it in 4 h (Fig. 8c). When glucose and cellobiose were added, PC-3-7 first assimilated glucose and after its depletion, cellobiose was assimilated slowly (Fig. 8d). On the other hand, PC-3-7Δ*tubB* assimilated glucose and cellobiose simultaneously, with glucose consumed more slowly than when only glucose was added. These results suggested that PC-3-7Δ*tubB* has a higher cellobiose uptake rate than the parent strain, and can uptake cellobiose even in the presence of glucose.

## Discussion

This study revealed that enzyme production was enhanced in a *tubB*-disrupted strain. Additionally, the deletion of *tubB* enabled high enzyme production in the presence of glucose. Cellobiose uptake was enhanced in the *tubB*-disrupted strain and cellobiose was taken up simultaneously with glucose, suggesting release from CCR. This is the first report that alpha-tubulin in *T. reesei* is involved in cellulase and hemicellulase gene expression, enzyme production, and CCR, and that CCR can be further released by genetic modification of *T. reesei*.

Microtubules formed by polymerization of tubulin proteins are not only involved in cell division, but also in vesicular trafficking. In *T. reesei*, it has been reported that disruption of *rho3* encoding ras-type small GTPase involved in cell polarity and vesicle fusion with the plasma membrane caused a reduction of secreted protein production [39]. However, there have been no reports that knockout of tubulin, which is involved in vesicular transport, improves the amount of proteins secreted by vesicular transport. So, this is the first report that deletion of tubulin enhances the secretory protein production in fungi. In this study, we confirmed that disruption of the *tubB* gene enhanced sugar transporter genes, and these membrane proteins are also transported by vesicular transport. Whether TubB is involved in the transport of extracellular proteins, transport of membrane proteins such as sugar transporters, or some other mechanism will require further molecular biological analysis of TubB.

*T. reesei* SEU-7, constructed by the overexpression of endogenous BGL in RUT-C30, exhibited a significant decrease in cellulase production by the addition of only 1% (w/v) glucose into medium containing cellulose [27]. This suggests that a high glucose concentration decreases cellulase production even in a Cre1-deficient strain, consistent with the extremely low protein production of PC-3-7 in glucose-containing medium (Fig. 5b). Several studies have investigated this decrease in enzyme production caused by glucose, aiming to further release CCR. Random mutagenesis released CCR in PC-3-7, although the molecular mechanism was not determined [40]. We found that disruption of the *tubB* gene enabled efficient enzyme production by utilizing cellulose in the presence of glucose. High protein production at a high glucose concentration cannot be achieved by knockout of *cre1* alone [27]. Thus, PC-3-7Δ*tubB* strain may be applicable to various *T. reesei* industrial enzyme production processes, such as fed-batch culture with insoluble inducers (such as cellulose and lignocellulose) and soluble sugars [41], fed-batch culture with glucose and soluble inducers [29], and fed-batch culture with glucose by gene modification of cellulase transcription activators [42].

High protein production, a characteristic of the tubulin-disrupted strain, was observed when the strain was cultured with only cellulose, cellulose and xylan, or cellulose and glucose. This strain also has a high production rate of enzyme. Figure 4 shows rapid enzyme production by E1AB1, PC-3-7Δ*tubB::Pegl1-aabgl1* and PC-3-7Δ*tubB*. Protein production by E1AB1 and PC-3-7Δ*tubB::Pegl1-aabgl1* was complete after 5 days, whereas PC-3-7Δ*tubB* continued to produce protein for 6 days, resulting in higher protein production than the other strains. The early cessation of protein production by E1AB1 and PC-3-7Δ*tubB::Pegl1-aabgl1* may be due to fast degradation of cellulose as a result of the secretion of AaBGL1 into the culture medium. On the other hand, PC-3-7Δ*tubB* does not produce AaBGL1 but its enzyme production rate is similar to that of E1AB1*.* Therefore, disruption of the *tubB* gene causes the increase in the rate of enzyme production regardless of the high expression of BGL. Figure 5d shows that PC-3-7Δ*tubB* generates more CO_2_ than PC-3-7 from the early stage of fermentation when cultured with cellulose, indicating that cellulose is efficiently degraded and assimilated by PC-3-7Δ*tubB*. Since PC-3-7Δ*tubB* can produce enzyme from the early stage of fermentation due to further release of CCR, PC-3-7Δ*tubB* can assimilate more sugars released from cellulose than PC-3-7, which may improve the rate of enzyme production.

PC-3-7Δ*tubB* has a higher uptake rate of cellobiose than PC-3-7 (Fig. 8). Sugar transporter genes are upregulated in PC-3-7Δ*tubB*, with cellobiose transporter ID 69957 being the most upregulated and cellobiose transporter *crt1* being the highest expressed transporter (Additional file 10: Table S7). In addition, when cultivated in a mixture of cellobiose and glucose, PC-3-7 did not decrease the concentration of cellobiose until glucose was depleted whereas PC-3-7Δ*tubB* decreased the cellobiose concentration (Fig. 8c, d). Moreover, when cultured with cellulose and glucose, no cellobiose could be detected in PC-3-7 until the glucose was depleted, whereas cellobiose was released similarly by PC-3-7Δ*tubB* as when cultivated with only cellulose (Fig. 5f, g). These results suggest that cellulase was produced even in the presence of glucose by PC-3-7Δ*tubB* and cellobiose was released from cellulose into the culture broth, but cellulase was not produced by PC-3-7. Furthermore, the high cellobiose uptake ability of PC-3-7Δ*tubB* even in the presence of glucose is evidence of its further resistance to CCR compared to PC-3-7. The ability of faster and de-repressed cellobiose uptake may eliminates the product inhibition of CBH by keeping the extracellular cellobiose concentration at low level (Fig. 5f), and reduces the excessive generation of glucose by uptake before cellobiose is degraded by BGL, leading to less CCR. In addition, faster uptake of cellobiose may lead to an increase in the intracellular cellobiose concentration, which may help keep cellulase induction condition by cellobiose. These three aspects may explain the high amount and rate of enzyme production by *tubB*-deleted strain.

The CAZyme genes upregulated by *tubB* deletion were *egl4* (gene ID: 73643), *xyn1*(74223), *xyn2* (123818), *xyn4* (111849), and *axe1* (73632) (Fig. 6, Additional file 5: Table S2). In addition, other genes such as *cip2* (123940), *bxl1* (121127), *aguA* (72526) and glycoside hydrolase family 27 (putative α-galactosidase, 72704) were also highly expressed in PC-3-7Δ*tubB* (Additional file 8: Table S5). Since all the expressed genes except for *egl4* are hemicellulases, deletion of the *tubB* gene may affect the induction of hemicellulases by cellulose.

In *T. reesei* QM9414, *xyn1* was induced by xylose and xylan but not by cellulose, whereas *xyn2* was induced by xylan and cellulose [43]. In PC-3-7, both *xyn1* and *xyn2* were induced by cellulose, xylose and xylan, but the expression of *xyn1* was lower than that of *xyn2* [44]. According to previous studies, the response of *xyn1* to cellulose changed following the mutation of QM9414 to PC-3-7. In PC-3-7Δ*tubB*, the expression level of *xyn1* induced by cellulose increased to the same level as that of *xyn3*, which was reported to be highly expressed in the presence of cellulose. The expression of *xyn1* was activated by Xyr1 and repressed by the transcription factors Cre1 and Ace1 [45]. However, in the present study, the expression of *cre1* and *ace1* was not significantly changed by *tubB* deletion, suggesting that the high expression of *xyn1* was not due to changes in the expression levels of these transcription factors. The upregulation of many CAZyme genes by *tubB* deletion indicates an unknown regulatory mechanism for these genes involving *tubB*.

## Conclusions

This study revealed that α_2_-tubulin in *T. reesei* is involved in the transcriptional regulation of cellulases and hemicellulases, CCR, cellobiose uptake, and protein production. Additionally, the α_2_-tubulin-deficient strain showed further resistance to CCR. This strain has the very useful characteristic of high resistance to CCR and high enzyme production even in the presence of glucose. Therefore, PC-3-7Δ*tubB* may be applicable to all *T. reesei* enzyme production processes utilizing glucose or cellulose as a carbon source. This study will help reduce the cost of enzyme production and aid the low cost production of sugar from cellulosic biomass.

## Methods

### Fungal strains and maintenance

The fungal strains are listed in Table 1. The strains were maintained on potato dextrose agar (PDA, Difco Laboratories, Detroit, MI) plates.

### Protein production

For preculture for enzyme production, 10^7^ conidia of each strain were inoculated into 50 mL of basal medium [24] containing 1% (w/v) glucose in a 500-mL Erlenmeyer flask. Conidia were counted using a TC20 Automated Cell Counter (Bio-Rad, Hercules, CA). The basal medium comprised 0.14% (w/v) (NH_4_)_2_SO_4_, 0.2% (w/v) KH_2_PO_4_, 0.03% (w/v) CaCl_2_·2H_2_O, 0.03% (w/v) MgSO_4_·7H_2_O, 0.1% (w/v) polypeptone, 0.05% (w/v) yeast extract, 0.1% (w/v) Tween 80 and 0.1% (w/v) trace element solution in 50 mM tartarate-Na buffer (pH 4.0). The trace element solution contained 6 mg of H_3_BO_3_, 26 mg of (NH_4_)_6_Mo_7_O_24_·4H_2_O, 100 mg of FeCl_3_·6H_2_O, 40 mg of CuSO_4_·5H_2_O, 8 mg of MnCl_2_·4H_2_O and 200 mg of ZnCI_2_ in 100 mL of distilled water. Precultures were carried out with shaking at 220 rpm and 28 °C for 2 days. The main cultures (1 L) of basal medium containing carbon sources such as microcrystalline cellulose (Avicel PH 101, Sigma–Aldrich, St. Louis, MO), xylan (beechwood xylan, Tokyo Chemical Industry Co. Ltd., Tokyo, Japan) and glucose (Wako Pure Chemical Industries, Ltd., Osaka, Japan) in a 2-L jar fermenter (BMZ-01KP2, Biott Co., Ltd., Tokyo, Japan) were inoculated with 100 mL of preculture. The main culture was carried out at 28 °C, with 0.5 volumes of air per volume of liquid per minute aeration and 3 ppm dissolved oxygen controlled by mechanical agitation. The pH was controlled at 4.5 using ammonia solution (5% w/v). An aliquot of the culture broth (2 mL) was collected every 24 h, cells were harvested by centrifugation at 20,000 × *g* for 5 min, then the supernatant was filtered through a 0.20-μm cellulose acetate membrane filter (13CP020AN; Advantec, Toyo Roshi Kaisha Ltd., Tokyo, Japan). The endpoint of the cultivation was the sampling point after the ammonia solution, which is a nitrogen source and used to keep the pH constant, was no longer supplied. The culture supernatants were analyzed to determine protein concentration using the Bradford protein assay (Bio-Rad) with bovine gamma globulin as the standard. The fermentations were carried out in triplicate. Student's t-test was used to compare the amount of protein production, and a *p* value less than 0.05 was considered statistically significant.

### Plate assay

To study the morphology of the *tubB*-deficient strains, transformants and parent strain were cultivated on PDA plate. The plates were grown at 30 °C for 4 days and the colony size (diameter) was measured for every day. The colony size was compared using Student's t-test.

### Scanning electron microscope (SEM) observation

SEM samples were prepared according to a previously reported method [46] with minor modifications. *T. reesei* was grown in 50 mL of basal medium containing 1% (w/v) glucose for 1 day or 1% (w/v) cellulose for 3 days in a 500-mL Erlenmeyer flask at 28 °C with shaking at 250 rpm. Hyphae were fixed in 2% (v/v) glutaraldehyde in 0.1 M cacodylate buffer, pH 7.4. Post-fixation procedures were conducted by Hanaichi Ultrastructure Research Institute Co., Ltd. (Aichi, Japan). For SEM image analysis, 70 cells were randomly chosen and cell length and diameter were measured using ImageJ (http://imagej.nih.gov/ij/). The cell length was measured from septum to septum. The length, diameter and ratio (length/diameter) of the cells were compared using Student's t-test.

### Cell growth analysis

For preculture, 10^7^ conidia of each strain were inoculated into 50 mL of basal medium containing 1% (w/v) glucose in a 500-mL Erlenmeyer flask. Main culture was started by inoculating 5 mL of precultures in the 50 mL of basal medium with 1%(w/v) glucose in a 500-mL Erlenmeyer flask. The culture was carried out with shaking at 220 rpm and 28 °C for 24 h. An aliquot of the culture broth (2 mL) was collected as appropriate, and cells were harvested by centrifugation at 20,000×*g* for 5 min, then the supernatant was filtered through a 0.20-μm cellulose acetate membrane filter (13CP020AN) and used for sugar analysis. Cells were washed twice with 1 mL of distilled water, and then the wet cell weight was measured.

### Sugar analyses

Glucose, xylose and cellobiose in the culture supernatants were analyzed according to a previously reported method [47] with minor modifications. The samples were diluted and analyzed on a LaChrom Elite HPLC System (Hitachi High-Technologies, Ibaraki, Japan) equipped with an ICSep-ION-300 column (Tokyo Chemical Industry Co. Ltd.). Separation was performed by isocratic elution with 5 mM H_2_SO_4_ at a flow rate of 0.5 mL/min for 30 min.

### SDS-PAGE analysis

SDS-PAGE was carried out with Any kD Mini-PROTEAN TGX Precast Protein Gels (Bio-Rad) for 35 min at 200 V. The gel was activated for 5 min and imaged using the ChemiDoc MP imaging system (Bio-Rad). The molecular weights of protein bands were estimated using Image Lab software (Bio-Rad). The molecular mass marker comprised 5 or 10 μL of Precision Plus Protein Unstained Standard (Bio-Rad).

### Identification of the locus of the inserted cassette by inverse PCR

Genomic DNA was extracted and purified using a modified version of the protocol from Seiboth et al. [48]. The genomic DNA was treated with the restriction enzymes XbaI and XhoI (Thermo Fisher Scientific**,** San Jose, CA) and then self-ligated using Ligation High (Toyobo, Osaka, Japan). Self-ligated DNAs were used as templates for PCR amplifications using the DNA polymerase PrimeSTAR Max (Takara Bio, Shiga, Japan). Amplified fragments were cloned and sequenced.

### Construction of plasmids for deletion of the *tubB* gene

The PCR primers are listed in Additional file 11: Table S8. Restriction sites for vector linearization and vector-specific extensions were introduced into these primers to facilitate cloning as required. The amplified fragments were ligated using an In-Fusion HD cloning kit (Takara Bio). Plasmids, primer pairs and templates used for construction of the plasmids are listed in Additional file 12: Table S9.

### Construction of pUC-Δ*tubB*-*amdS*

The promoter, ORF, transcription terminator, and 3′ flanking sequences of *T. reesei cbh1* were amplified from the genomic DNA of *T. reesei* strain PC-3-7 using the primers swaI *tubB* F and swaI *tubB* R. A vector fragment was amplified by inverse PCR using pUC118 (Takara Bio) as the template with the primers swaI pUC F and swaI pUC R. The amplified fragments were ligated using an In-Fusion HD cloning kit to yield pUC-*tubB.*

The gene *amdS* (encoding an *A. nidulans* acetamidase gene) was amplified from pUC-*amdS* using the primers *amdS* F and *amdS* R [49]. The fragment generated by inverse PCR using pUC-*tubB* with the primers *tubBback*-*amdS* F and *tubBfront*-*amdS* R was fused to the *amdS* fragment to generate pUC-Δ*tubB*-*amdS*.

### Construction of pUC-Δ*tubB*-*Pegl1-aabgl1-amdS*

The *aabgl1* expression cassette with the *amdS* marker containing the *egl1* promoter, the ORF of *aabgl1,* and the transcription terminator and 3′ flanking sequences of *egl1* and *amdS* marker were amplified from the genomic DNA of *T. reesei* strain E1AB1 using the PCR primers *tubBfront*-*Pegl1* F and *amdS* R. A vector fragment was amplified by inverse PCR using pUC-*tubB* with the primers *tubBback-amdS* F and *tubBfront* R. The obtained fragments were ligated to yield pUC-Δ*tubB*-*Pegl1-aabgl1-amdS.*

### Transformation of *T. reesei*

Plasmids were linearized with *Swa*I prior to transformation. *T. reesei* was transformed using the protoplast-PEG method [49] modified to use Yatalase (Takara Bio) as a protoplasting enzyme instead of Novozyme 234 (Novozymes, Bagsværd, Denmark). Transformed protoplasts were plated on transformation medium [2.0% (w/v) glucose, 18.27% (w/v) sorbitol, 0.06% (w/v) acetamide, 0.2% (w/v) CaCl_2_, 0.06% (w/v) MgSO_4_, 0.21% (w/v) CsCl and 0.1% (w/v) trace element solution in 100 mM KH_2_PO_4_ buffer (pH 5.5)]. The trace element solution contained 500 mg of FeSO_4_·7H_2_O, 200 mg of CoCl_2_, 160 mg of MnSO_4_·H_2_O and 140 mg of ZnSO_4_·7H_2_O in 100 mL of distilled water. After 2 weeks’ incubation at 28 °C, candidate transformants were streaked twice on selective plates (transformation medium without sorbitol) for several days at 28 °C to isolate single colonies, then single colonies were transferred to PDA plates to form conidia for 1 week at 28 °C. One transformant was confirmed by colony PCR using KOD FX Neo (Toyobo) according to the manufacturer's protocol.

### Enzyme assay

Cellulase activities were measured as pNPGase for BGL activity, pNPLase for CBH1 activity, and CMCase for EG activity. Hemicellulase activities were measured as pNPXase for BXL activity and pNPX2ase and xylanase for XYN activity. Enzymatic activity against synthetic substrate was measured by assaying the *p*-nitrophenol released from 1 mM *p*-nitrophenyl glucopyranoside (pNPG, Sigma-Aldrich), *p*-nitrophenyl lactoside (pNPL, Sigma-Aldrich), *p*-nitrophenyl xyloside (pNPX, Sigma-Aldrich), and *p*-nitrophenyl xylobioside (pNPX2, Megazyme) in 50 mM sodium acetate buffer (pH 5.0) at 50 °C for 10 min. Reactions were performed in a total volume of 0.1 mL and stopped by adding an equal volume of 0.4 M NaHCO_3_. The released *p*-nitrophenol was quantified by measuring the absorbance at 420 nm. One unit of activity was defined as the amount of enzyme that produced 1 μmol of *p*-nitrophenol per minute from the substrate. CMCase and xylanase activities were determined with 1%(w/v) carboxymethyl cellulose (Tokyo Chemical Industry) and 1%(w/v) beechwood xylan (Tokyo Chemical Industry) in 50 mM sodium acetate buffer (pH 5.0) at 50 °C for 10 min. Reactions were performed in a total volume of 0.1 mL and stopped by adding an equal volume of alkaline 3,5-dinitrosalicylic (DNS) and heating for 5 min at 100 °C [50]. Then the absorbance at 540 nm was detected. One unit of activity was defined as the amount of enzyme that produced 1 μmol of reducing sugar per minute in glucose equivalents.

### RNA-Seq

Total RNA extraction and cDNA synthesis were performed using an RNeasy Plant Mini Kit (Qiagen, Crawley, UK) according to the manufacturer's instructions. Total RNA was employed for next-generation sequencing libraries using a TruSeq RNA Sample Prep Kit (Illumina, San Diego, CA). Libraries were sequenced using the Illumina MiSeq platform. The obtained sequences were quality-filtered by CLC Genomics Workbench using the default settings. Filtered reads were mapped to *T. reesei* QM6a cds sequences downloaded from the Ensembl Fungi database (https://fungi.ensembl.org/Trichoderma_reesei/Info/Index/) by CLC Genomics Workbench using the default settings. The raw count matrix consisting of 9,115 genes × 24 samples (= 2 strains × 2 sampling times × 2 media × 3 biological replicates) was obtained. The RPKM of each gene was calculated by CLC Genomics Workbench using the default settings. The raw read count matrix for every gene and every sample was shown in Additional file 13: Table S10. RPKM values for every gene for each sample was shown in Additional file 14: Table S11.

### Differentially expressed gene (DEG) analysis

Normalization of the raw count matrix and identification of the DEGs was performed using R (ver. 3.6.1, R Core Team (2018). R: A language and environment for statistical computing. R Foundation for Statistical Computing, Vienna, Austria. URL https://www.R-project.org/.) and DESeq2 package [51]. Genes for which the adjusted *p* < 0.01 were identified as statistically significant DEGs.

### Gene ontology (GO) enrichment analysis of DEGs

GO enrichment analysis was implemented by using the R and GOseq packages based on Wallenius non-central hyper-geometric distribution [52], which can adjust for gene length bias in DEGs. GO annotations of each gene were obtained from the JGI database (http://genome.jgi.doe.gov/Trire2/Trire2.home.html). GO terms with over-represented *p* < 0.01 were considered significantly enriched in DEGs.

## Supplementary Information

**Additional file 1: Figure S1.** Maps of the aabgl1-expression cassette integrated in the *T. reesei* E1AB1 and PC-3-7 genomes. (a) Maps of the genome around the *tubB* gene (Gene ID: 120830) of PC-3-7 and E1AB1 are shown on the top and bottom, respectively. The arrows indicate the gene and its direction of transcription and the squares indicate the promoter and terminator region. Blue, green, yellow and orange indicate the sequences around *tubB*, *egl1*, *amdS* and *aabgl1*, respectively. Scale bar indicates 1 kb. The sites where the primers used to determine the inserted locus could anneal are marked with black arrows. (b) Identification of inserted locus by PCR amplification. At the top of each lane, the genome used as template and the primer number used are indicated. M in the lane indicates marker (Gene Ladder Wide 2, Nippon Gene, Toyama, Japan).

**Additional file 2: Figure S2**. Cell growth and glucose consumption of PC-3-7, E1AB1 and PC-3-7Δ*tubB* in the basal medium with glucose. Culture was started by inoculating 5 mL of precultures in the 50 mL of basal medium with 1%(w/v) glucose in a 500-mL Erlenmeyer flask. The culture was carried out with shaking at 220 rpm and 28°C for 24 hours. Blue line indicates PC-3-7, orange line indicates E1AB1 and gray color indicates PC-3-7Δ*tubB*. **a** Wet cell weight. **b** Glucose concentration in the culture broth. Data are expressed as mean ± SD of three biological replicates.

**Additional file 3: Figure S3.** Specific enzyme activity and SDS-PAGE analysis of the produced protein. **a** Specific activity of cellulolytic and hemicellulolytic enzyme of PC-3-7 and PC-3-7Δ*tubB* when cultivated in medium with 10% (w/v) microcrystalline cellulose or 10% (w/v) microcrystalline cellulose and 2.5% (w/v) glucose. Data are expressed as mean ± SD of three biological replicates. SDS-PAGE analysis loading 1 μL of each culture supernatant after 120 hours of cultivation (**b**) and loading 5 μg of each protein amount (c). The molecular mass marker comprised 5 μL (**c**) or 10 μL (**d**) of Precision Plus Protein Unstained Standard (Bio-Rad). Lanes: Precision Plus Protein Unstained Standard (M); supernatant of PC-3-7 cultivated in the medium with 10% (w/v) microcrystalline cellulose (1) and cultivated in the medium with 10% (w/v) microcrystalline cellulose and 2.5% (w/v) glucose (2); supernatant of PC-3-7Δ*tubB* cultivated in the medium with 10% (w/v) microcrystalline cellulose (3) and cultivated in the medium with 10% (w/v) microcrystalline cellulose and 2.5% (w/v) glucose (4).

**Additional file 4: Table S1.** RPKM values of tubulins.

**Additional file 5: Table S2. **RPKMs of major CAZymes.

**Additional file 6: Table S3.** RPKM values of transcription factors.

**Additional file 7: Table S4.** Correlation matrix of RPKMs between all RNA-seq conditions.

**Additional file 8: Table S5.** CAZymes that were significantly changed in the PC-3-7Δ*tubB* compared to PC-3-7.

**Additional file 9: Table S6. **Enriched gene ontology categories of DEGs between PC-3-7 and PC-3-7Δ*tubB*.

**Additional file 10: Table S7.** The differentially expressed predicted sugar transporter genes.

**Additional file 11: Table S8.** PCR primers.

**Additional file 12: Table S9.** Plasmids, PCR templates and primers used for plasmid construction.

**Additional file 13: Table S10.** Matrix table with raw read counts.

**Additional file 14: Table S11.** RPKM values for every gene.

## Data Availability

The datasets used and/or analyzed during the current study are included in this published article and its supplemental information files.

## Abbreviations

BGL: Beta-glucosidase
BXL: Beta-xylosidase
CAZyme: Carbohydrate-active enzyme
CBH: Cellobiohydrolase
CCR: Carbon catabolite repression
CDS: Coding sequence
CMC: Carboxymethyl cellulose
DEG: Differentially expressed gene
EG: Endoglucanase
GO: Gene ontology
ORF: Open reading frame
pNPG: *p*-Nitrophenyl glucopyranoside
pNPL: *p*-Nitrophenyl lactoside
pNPX: *p*-Nitrophenyl xyloside
pNPX2: *p*-Nitrophenyl xylobioside
SEM: Scanning electron microscope
XYN: Endoxylanase

## References

[1] Farrell AE, Plevin RJ, Turner BT, Jones AD, O’Hare M, Kammen DM (2006). Ethanol can contribute to energy and environmental goals. Science.

[2] Nagesh M. Industrial Enzymes Market by Type (Carbohydrases, Proteases, Lipases, Polymerases & Nucleases, Other Types), Source, Application (Food & Beverages, Feed, Bioethanol, Detergents, Pulp & Paper, Textiles & Leather, Wastewater Treatment, Other Applications), Form, and Region - Global Forecast to 2026. MarketsandMarkets; 2020.

[3] Martinez D, Berka RM, Henrissat B, Saloheimo M, Arvas M, Baker SE (2008). Genome sequencing and analysis of the biomass-degrading fungus Trichoderma reesei (syn. Hypocrea jecorina). Nature Biotechnol..

[4] Brady SK, Sreelatha S, Feng Y, Chundawat SPS, Lang MJ (2015). Cellobiohydrolase 1 from *Trichoderma reesei* degrades cellulose in single cellobiose steps. Nature Commun.

[5] Castro L dos S, Pedersoli WR, Antoniêto ACC, Steindorff AS, Silva-Rocha R, Martinez-Rossi NM, et al. Comparative metabolism of cellulose, sophorose and glucose in *Trichoderma reesei* using high-throughput genomic and proteomic analyses. Biotechnol. Biofuels. 2014;7:41. 10.1186/1754-6834-7-41 PMC3998047 24655731

[6] Kubicek CP, Mikus M, Schuster A, Schmoll M, Seiboth B (2009). Metabolic engineering strategies for the improvement of cellulase production by *Hypocrea jecorina*. Biotechnol Biofuels.

[7] Himmel ME, Ding S-Y, Johnson DK, Adney WS, Nimlos MR, Brady JW (2007). Biomass recalcitrance: Engineering plants and enzymes for biofuels production. Science.

[8] Lin Y, Tanaka S (2006). Ethanol fermentation from biomass resources: current state and prospects. Appl Microbiol Biotechnol.

[9] Schuster A, Schmoll M (2010). Biology and biotechnology of *Trichoderma*. Appl Microbiol Biotechnol.

[10] Bischof RH, Ramoni J, Seiboth B (2016). Cellulases and beyond: the first 70 years of the enzyme producer *Trichoderma reesei*. Microb Cell Fact.

[11] Saloheimo M, Kuja-Panula J, Ylösmäki E, Ward M, Penttilä M (2002). Enzymatic properties and intracellular localization of the novel *Trichoderma reesei* β-glucosidase BGLII (Cel1A). Appl Environ Microbiol.

[12] Gruno M, Väljamäe P, Pettersson G, Johansson G (2004). Inhibition of the *Trichoderma reesei* cellulases by cellobiose is strongly dependent on the nature of the substrate. Biotechnol Bioeng.

[13] Barnett CC, Berka RM, Fowler T (1991). Cloning and amplification of the gene encoding an extracellular β-glucosidase from *Trichoderma reesei*: Evidence for improved rates of saccharification of cellulosic substrates. Bio/Technology.

[14] Zhang J, Zhong Y, Zhao X, Wang T (2010). Development of the cellulolytic fungus *Trichoderma reesei* strain with enhanced β-glucosidase and filter paper activity using strong artificial cellobiohydrolase 1 promoter. Bioresour Technol.

[15] Nakazawa H, Kawai T, Ida N, Shida Y, Kobayashi Y, Okada H (2012). Construction of a recombinant *Trichoderma reesei* strain expressing *Aspergillus aculeatus* β-glucosidase 1 for efficient biomass conversion. Biotechnol Bioeng.

[16] Nakazawa H, Kawai T, Ida N, Shida Y, Shioya K, Kobayashi Y (2016). A high performance *Trichoderma reesei* strain that reveals the importance of xylanase III in cellulosic biomass conversion. Enzyme Microb Technol.

[17] Stricker AR, Grosstessner-Hain K, Würleitner E, Mach RL (2006). Xyr1 (xylanase regulator 1) regulates both the hydrolytic enzyme system and D-xylose metabolism in *Hypocrea jecorina*. Eukaryot Cell.

[18] Aro N, Ilmén M, Saloheimo A, Penttilä M (2003). ACEI of *Trichoderma reesei* is a repressor of cellulase and xylanase expression. Appl Environ Microbiol.

[19] Aro N, Saloheimo A, Ilmén M, Penttilä M (2001). ACEII, a novel transcriptional activator involved in regulation of cellulase and xylanase genes of *Trichoderma reesei*. J Biol Chem.

[20] Häkkinen M, Valkonen MJ, Westerholm-Parvinen A, Aro N, Arvas M, Vitikainen M (2014). Screening of candidate regulators for cellulase and hemicellulase production in *Trichoderma reesei* and identification of a factor essential for cellulase production. Biotechnol Biofuels.

[21] Strauss J, Mach RL, Zeilinger S, Hartler G, Stöffler G, Wolschek M (1995). Crel, the carbon catabolite repressor protein from *Trichoderma reesei*. FEBS Lett.

[22] Montenecourt BS, Eveleigh DE (1979). Screening methods for the isolation of high yielding cellulase mutants of *Trichoderma reesei*.

[23] Nakari-Setälä T, Paloheimo M, Kallio J, Vehmaanperä J, Penttilä M, Saloheimo M (2009). Genetic modification of carbon catabolite repression in *Trichoderma reesei* for improved protein production. Appl Environ Microbiol.

[24] Kawamori M, Morikawa Y, Takasawa S (1986). Induction and production of cellulases by l-sorbose in *Trichoderma reesei*. Appl Microbiol Biotechnol.

[25] Porciuncula J de O, Furukawa T, Mori K, Shida Y, Hirakawa H, Tashiro K, et al. Single nucleotide polymorphism analysis of a *Trichoderma reesei* hyper-cellulolytic mutant developed in Japan. Biosci Biotechnol Biochem 2013;77:534–43 10.1271/bbb.120794 23470758

[26] Ilmén M, Thrane C, Penttilä M (1996). The glucose repressor gene *cre1* of *Trichoderma*: Isolation and expression of a full-length and a truncated mutant form. Molec Gen Genet.

[27] Li C, Lin F, Zhou L, Qin L, Li B, Zhou Z (2017). Cellulase hyper-production by *Trichoderma reesei* mutant SEU-7 on lactose. Biotechnol Biofuels.

[28] Jourdier E, Cohen C, Poughon L, Larroche C, Monot F, Chaabane FB (2013). Cellulase activity mapping of *Trichoderma reesei* cultivated in sugar mixtures under fed-batch conditions. Biotechnol Biofuels.

[29] Li Y, Liu C, Bai F, Zhao X (2016). Overproduction of cellulase by T*richoderma reesei* RUT C30 through batch-feeding of synthesized low-cost sugar mixture. Bioresour Technol.

[30] Desai A, Mitchison TJ (1997). Microtubule polymerization dynamics. Annu Rev Cell Dev Biol.

[31] Zhao Z, Liu H, Luo Y, Zhou S, An L, Wang C (2014). Molecular evolution and functional divergence of tubulin superfamily in the fungal tree of life. Sci Rep.

[32] Doshi P, Bossie CA, Doonan JH, Mays GS, Morris NR (1991). Two α-tubulin genes of *Aspergillus nidulans* encode divergent proteins. Molec Gen Genet.

[33] Kirk KE, Morris NR (1993). Either alpha-tubulin isogene product is sufficient for microtubule function during all stages of growth and differentiation in *Aspergillus nidulans*. Mol Cell Biochem.

[34] Nogueira KMV, de Paula RG, Antoniêto ACC, dos Reis TF, Carraro CB, Silva AC (2018). Characterization of a novel sugar transporter involved in sugarcane bagasse degradation in *Trichoderma reesei*. Biotechnol Biofuels.

[35] Zhang W, Kou Y, Xu J, Cao Y, Zhao G, Shao J (2013). Two major facilitator superfamily sugar transporters from *Trichoderma reesei* and their roles in induction of cellulase biosynthesis. J Biol Chem.

[36] Havukainen S, Valkonen M, Koivuranta K, Landowski CP (2020). Studies on sugar transporter CRT1 reveal new characteristics that are critical for cellulase induction in *Trichoderma reesei*. Biotechnol Biofuels.

[37] Huang Z-B, Chen X-Z, Qin L-N, Wu H-Q, Su X-Y, Dong Z-Y (2015). A novel major facilitator transporter TrSTR1 is essential for pentose utilization and involved in xylanase induction in *Trichoderma reesei*. Biochem Biophys Res Commun.

[38] Porciuncula J de O, Furukawa T, Shida Y, Mori K, Kuhara S, Morikawa Y, et al. Identification of major facilitator transporters involved in cellulase production during lactose culture of *Trichoderma reesei* PC-3-7. Biosci Biotechnol Biochem. 2013;77:1014–22. 10.1271/bbb.120992 23649266

[39] Vasara T, Salusjärvi L, Raudaskoski M, Keränen S, Penttilä M, Saloheimo M (2001). Interactions of the Trichoderma reesei rho3 with the secretory pathway in yeast and *T. reesei*. Mol Microbiol.

[40] Ike M, Park J, Tabuse M, Tokuyasu K (2010). Cellulase production on glucose-based media by the UV-irradiated mutants of *Trichoderma reesei*. Appl Microbiol Biotechnol.

[41] Ellilä S, Fonseca L, Uchima C, Cota J, Goldman GH, Saloheimo M (2017). Development of a low-cost cellulase production process using *Trichoderma reesei* for Brazilian biorefineries. Biotechnol Biofuels.

[42] Zhang X, Li Y, Zhao X, Bai F (2017). Constitutive cellulase production from glucose using the recombinant *Trichoderma reesei* strain overexpressing an artificial transcription activator. Bioresour Technol.

[43] Zeilinger S, Mach RL, Schindler M, Herzog P, Kubicek CP (1996). Different inducibility of expression of the two xylanase genes *xyn1* and *xyn2* in *Trichoderma reesei*. J Biol Chem.

[44] Xu J, Nogawa M, Okada H, Morikawa Y (2000). Regulation of *xyn3* gene expression in *Trichoderma reesei* PC-3–7. Appl Microbiol Biotechnol.

[45] Rauscher R, Würleitner E, Wacenovsky C, Aro N, Stricker AR, Zeilinger S (2006). Transcriptional regulation of *xyn1*, encoding xylanase I, in *Hypocrea jecorina*. Eukaryot Cell.

[46] Nykänen M, Birch D, Peterson R, Yu H, Kautto L, Gryshyna A (2016). Ultrastructural features of the early secretory pathway in *Trichoderma reesei*. Curr Genet.

[47] Shi N-Q, Cruz J, Sherman F, Jeffries TW (2002). SHAM-sensitive alternative respiration in the xylose-metabolizing yeast *Pichia stipitis*. Yeast.

[48] Seiboth B, Hartl L, Pail M, Fekete E, Karaffa L, Kubicek CP (2004). The galactokinase of *Hypocrea jecorina* is essential for cellulase induction by lactose but dispensable for growth on d-galactose. Mol Microbiol.

[49] Penttilä M, Nevalainen H, Rättö M, Salminen E, Knowles J (1987). A versatile transformation system for the cellulolytic filamentous fungus *Trichoderma reesei*. Gene.

[50] Miller GL (1959). Modified DNS method for reducing sugars. Anal Chem.

[51] Love MI, Huber W, Anders S (2014). Moderated estimation of fold change and dispersion for RNA-seq data with DESeq2. Genome Biol.

[52] Young MD, Wakefield MJ, Smyth GK, Oshlack A (2010). Gene ontology analysis for RNA-seq: accounting for selection bias. Genome Biol.

