# Supplementary material for: Disruption of alpha-tubulin releases carbon catabolite repression and enhances enzyme production in Trichoderma reesei even in the presence of glucose
Source: Biotechnol Biofuels. 2021 Feb 8;14:39. doi: 10.1186/s13068-021-01887-0 (PMC7869464; doi:10.1186/s13068-021-01887-0)
Supplement: Supplementary file 1 — Additional file 1: Figure S1. Maps of the aabgl1-expression cassette integrated in the T. reesei E1AB1 and PC-3-7 genomes. (a) Maps of the genome around the tubB gene (Gene ID: 120830) of PC-3-7 and E1AB1 are shown on the top and bottom, respectively. The arrows indicate the gene and its direction of transcription and the squares indicate the promoter and terminator region. Blue, green, yellow and orange indicate the sequences around tubB, egl1, amdS and aabgl1, respectively. Scale bar indicates 1 kb. The sites where the primers used to determine the inserted locus could anneal are marked with black arrows. (b) Identification of inserted locus by PCR amplification. At the top of each lane, the genome used as template and the primer number used are indicated. M in the lane indicates marker (Gene Ladder Wide 2, Nippon Gene, Toyama, Japan). [file 13068_2021_1887_MOESM1_ESM.docx]

# Table S2: RPKMs of major CAZymes

| Gene ID | Gene  name | PC-3-7_ 24h_C | Δ*tubB* _ 24h_C | PC-3-7_ 24h_C+G | Δ*tubB* _ 30h_C+G | PC-3-7_ 48h_C | Δ*tubB* _ 48h_C | PC-3-7_ 48h_C+G | Δ*tubB* _ 48h_C+G |
| --- | --- | --- | --- | --- | --- | --- | --- | --- | --- |
| 123989 | *cbh1* | 75689 | 91412 | 31164 | 53634 | 57811 | 108728 | 9195 | 85791 |
| 72567 | *cbh2* | 43607 | 42204 | 19782 | 27310 | 32395 | 44940 | 6422 | 32675 |
| 120961 | *egl7* | 30599 | 20266 | 7158 | 7759 | 34346 | 23925 | 3694 | 19782 |
| 123232 | *egl3* | 18877 | 13673 | 4064 | 6062 | 16963 | 18535 | 2077 | 12052 |
| 123818 | *xyn2* | 16865 | 19063 | 5632 | 10873 | 12173 | 31079 | 2960 | 19403 |
| 120312 | *egl2* | 16497 | 13950 | 5994 | 7688 | 11797 | 12657 | 2133 | 9818 |
| 120229 | *xyn3* | 15215 | 12871 | 3302 | 4060 | 7612 | 11118 | 1218 | 7312 |
| 122081 | *egl1* | 15044 | 11607 | 6823 | 8120 | 11506 | 11918 | 2195 | 8802 |
| 73638 | *cip1* | 14195 | 12775 | 7427 | 12109 | 9718 | 13449 | 2233 | 12873 |
| 123992 | *swo* | 10023 | 10004 | 5501 | 6998 | 7715 | 11758 | 1383 | 8681 |
| 49976 | *egl5* | 6768 | 6423 | 4040 | 5413 | 6039 | 8492 | 1057 | 8462 |
| 73643 | *egl4* | 5688 | 11424 | 5449 | 10586 | 4003 | 13683 | 1129 | 14906 |
| 73632 | *axe1* | 5213 | 5846 | 4328 | 6460 | 2248 | 6899 | 1057 | 5880 |
| 120749 | *cel1a* | 3697 | 2712 | 3461 | 2757 | 3245 | 2714 | 1099 | 2633 |
| 112392 | *xyn4* | 2077 | 3347 | 250 | 2770 | 1410 | 3264 | 332 | 2465 |
| 56996 | *man5a* | 1043 | 2292 | 1861 | 1801 | 758 | 2748 | 254 | 2797 |
| 74223 | *xyn1* | 361 | 9786 | 538 | 8852 | 71 | 10787 | 8 | 7882 |
