# Supplementary material for: Disruption of alpha-tubulin releases carbon catabolite repression and enhances enzyme production in Trichoderma reesei even in the presence of glucose
Source: Biotechnol Biofuels. 2021 Feb 8;14:39. doi: 10.1186/s13068-021-01887-0 (PMC7869464; doi:10.1186/s13068-021-01887-0)
Supplement: Supplementary file 2 — Additional file 2: Figure S2. Cell growth and glucose consumption of PC-3-7, E1AB1 and PC-3-7ΔtubB in the basal medium with glucose. Culture was started by inoculating 5 mL of precultures in the 50 mL of basal medium with 1%(w/v) glucose in a 500-mL Erlenmeyer flask. The culture was carried out with shaking at 220 rpm and 28°C for 24 hours. Blue line indicates PC-3-7, orange line indicates E1AB1 and gray color indicates PC-3-7ΔtubB. a Wet cell weight. b Glucose concentration in the culture broth. Data are expressed as mean ± SD of three biological replicates. [file 13068_2021_1887_MOESM2_ESM.docx]

# Table S3. RPKMs of transcription factors

| Gene ID | Gene name | PC-3-7_  24h_C | Δ*tubB* _  24h_C | PC-3-7_  24h_C+G | Δ*tubB* 24h_C+G | PC-3-7_  48h_C | Δ*tubB* _  48h_C | PC-3-7_  48h_C+G | Δ*tubB* _  48h_C+G |
| --- | --- | --- | --- | --- | --- | --- | --- | --- | --- |
| 75418 | *ace1* | 60 | 47 | 133 | 79 | 79 | 54 | 171 | 78 |
| 78445 | *ace2* | 23 | 22 | 44 | 33 | 23 | 18 | 27 | 29 |
| 77513 | *ace3* | 328 | 393 | 642 | 328 | 270 | 411 | 125 | 393 |
| 52368 | *bglR* | 74 | 67 | 173 | 118 | 90 | 81 | 129 | 111 |
| 27600 | *clr1* | 8 | 10 | 16 | 11 | 8 | 11 | 10 | 10 |
| 26163 | *clr2* | 4 | 9 | 10 | 7 | 4 | 8 | 1 | 9 |
| 120117 | *cre1* | 118 | 168 | 271 | 356 | 145 | 244 | 261 | 386 |
| 124286 | *hap2* | 6 | 5 | 13 | 5 | 12 | 4 | 16 | 6 |
| 41617 | *lae1* | 5 | 3 | 8 | 8 | 7 | 4 | 20 | 7 |
| 72611 | *rce1* | 3 | 3 | 17 | 1 | 3 | 2 | 1 | 2 |
| 122284 | *vel1* | 27 | 16 | 48 | 20 | 37 | 23 | 47 | 25 |
| 54675 | *vib1* | 73 | 34 | 99 | 72 | 149 | 65 | 179 | 79 |
| 122208 | *xyr1* | 368 | 348 | 528 | 383 | 303 | 321 | 109 | 438 |
