# Supplementary material for: Disruption of alpha-tubulin releases carbon catabolite repression and enhances enzyme production in Trichoderma reesei even in the presence of glucose
Source: Biotechnol Biofuels. 2021 Feb 8;14:39. doi: 10.1186/s13068-021-01887-0 (PMC7869464; doi:10.1186/s13068-021-01887-0)
Supplement: Supplementary file 3 — Additional file 3: Figure S3. Specific enzyme activity and SDS-PAGE analysis of the produced protein. a Specific activity of cellulolytic and hemicellulolytic enzyme of PC-3-7 and PC-3-7ΔtubB when cultivated in medium with 10% (w/v) microcrystalline cellulose or 10% (w/v) microcrystalline cellulose and 2.5% (w/v) glucose. Data are expressed as mean ± SD of three biological replicates. SDS-PAGE analysis loading 1 μL of each culture supernatant after 120 hours of cultivation (b) and loading 5 μg of each protein amount (c). The molecular mass marker comprised 5 μL (c) or 10 μL (d) of Precision Plus Protein Unstained Standard (Bio-Rad). Lanes: Precision Plus Protein Unstained Standard (M); supernatant of PC-3-7 cultivated in the medium with 10% (w/v) microcrystalline cellulose (1) and cultivated in the medium with 10% (w/v) microcrystalline cellulose and 2.5% (w/v) glucose (2); supernatant of PC-3-7ΔtubB cultivated in the medium with 10% (w/v) microcrystalline cellulose (3) and cultivated in the medium with 10% (w/v) microcrystalline cellulose and 2.5% (w/v) glucose (4). [file 13068_2021_1887_MOESM3_ESM.docx]

# Table S4: Correlation matrix of RPKMs between all RNA-seq conditions

|  | PC-3-7_  24h_C | PC-3-7_  24h_C+G | PC-3-7_  48h_C | PC-3-7_  48h_C+G | Δ*tubB* _  24h_C | Δ*tubB* _  30h_C+G | Δ*tubB* _  48h_C | Δ*tubB* _  48h_C+G |
| --- | --- | --- | --- | --- | --- | --- | --- | --- |
| PC-3-7_24h_C | 1.000 |  |  |  |  |  |  |  |
| PC-3-7_24h_C+G | 0.894 | 1.000 |  |  |  |  |  |  |
| PC-3-7_48h_C | 0.977 | 0.895 | 1.000 |  |  |  |  |  |
| PC-3-7_48h_C+G | 0.505 | 0.733 | 0.597 | 1.000 |  |  |  |  |
| Δ*tubB* _24h_C | 0.978 | 0.891 | 0.938 | 0.478 | 1.000 |  |  |  |
| Δ*tubB* _30h_C+G | 0.908 | 0.951 | 0.904 | 0.704 | 0.931 | 1.000 |  |  |
| Δ*tubB* _48h_C | 0.966 | 0.872 | 0.938 | 0.47 | 0.989 | 0.925 | 1.000 |  |
| Δ*tubB* _48h_C+G | 0.952 | 0.899 | 0.942 | 0.551 | 0.974 | 0.956 | 0.988 | 1.000 |
