# Supplementary material for: Disruption of alpha-tubulin releases carbon catabolite repression and enhances enzyme production in Trichoderma reesei even in the presence of glucose
Source: Biotechnol Biofuels. 2021 Feb 8;14:39. doi: 10.1186/s13068-021-01887-0 (PMC7869464; doi:10.1186/s13068-021-01887-0)
Supplement: Supplementary file 4 — Additional file 4: Table S1. RPKM values of tubulins. [file 13068_2021_1887_MOESM4_ESM.docx]

# Table S5. CAZymes that were significantly changed in the PC-3-7Δ*tubB* compared to PC-3-7

| Gene ID | Gene  name | Enzyme | RPKM | | Log_2_ Fold Change of RPMK | FDR |
| --- | --- | --- | --- | --- | --- | --- |
|  |  | Family | PC-3-7_48h_C | Δ*tubB* _48h_C |  |  |
| 72526 | *aguA* | GH67 | 57 | 545 | 3.25 | 2.2E-70 |
| 72704 |  | GH27 | 26 | 232 | 3.14 | 6.1E-59 |
| 73643 | *egl4* | GH61 | 4003 | 13683 | 1.77 | 3.1E-54 |
| 54219 |  | CE5 | 8 | 339 | 5.40 | 8.2E-50 |
| 73632 | *axe1* | CE5 | 2248 | 6899 | 1.62 | 5.7E-38 |
| 69944 |  | GH31 | 15 | 98 | 2.75 | 6.7E-38 |
| 46816 | *cel3d* | GH3 | 25 | 107 | 2.12 | 8.3E-38 |
| 123456 |  | GH65 | 9 | 60 | 2.68 | 2.5E-37 |
| 123940 | *cip2* | CE15 | 662 | 1864 | 1.49 | 5.2E-34 |
| 111849 | *xyn4* | GH30 | 620 | 1969 | 1.67 | 4.6E-32 |
| 123818 | *xyn2* | GH11 | 12173 | 31079 | 1.35 | 6.1E-32 |
| 121127 | *bxl1* | GH3 | 569 | 1407 | 1.31 | 3.4E-28 |
| 56996 | *man5a* | GH5 | 758 | 2748 | 1.86 | 3.2E-25 |
| 55319 |  | GH54 | 8 | 60 | 2.86 | 3.9E-22 |
| 103049 | *pec2* | GH28 | 3 | 52 | 4.04 | 3.8E-20 |
| 57857 |  | GH2 | 26 | 68 | 1.37 | 1.1E-19 |
| 108671 |  | GH3 | 38 | 111 | 1.53 | 1.2E-19 |
| 5836 |  | GH2 | 19 | 51 | 1.43 | 3.1E-17 |
| 69276 |  | GH30 | 435 | 895 | 1.04 | 2.2E-16 |
| 27395 |  | GH76 | 17 | 66 | 1.95 | 4.1E-16 |
| 58282 |  | CE9 | 19 | 46 | 1.25 | 1.0E-14 |
| 23346 | *nag2* | GH20 | 121 | 23 | -2.41 | 8.0E-14 |
| 76210 |  | GH62 | 232 | 415 | 0.84 | 9.1E-14 |
| 58802 |  | GH95 | 1 | 17 | 4.73 | 1.1E-13 |
| 123989 | *cbh1* | GH7 | 57811 | 108728 | 0.91 | 2.3E-13 |
| 112392 |  | GH11 | 1410 | 3264 | 1.21 | 3.8E-13 |
| 76672 | *cel3a* | GH3 | 143 | 330 | 1.20 | 4.6E-12 |
| 74198 |  | GH92 | 3 | 16 | 2.33 | 7.3E-12 |
| 123538 | *gel1* | GH72 | 208 | 31 | -2.74 | 3.2E-11 |
| 72567 | *cbh2* | GH6 | 32395 | 44940 | 0.47 | 1.3E-10 |
| 120873 |  | GH71 | 52 | 92 | 0.83 | 1.5E-10 |
| 74223 | *xyn1* | GH11 | 71 | 10787 | 7.24 | 3.5E-10 |
| 73638 | *cip1* | - | 9718 | 13449 | 0.47 | 3.6E-10 |
| 49976 | *egl5* | GH45 | 6039 | 8492 | 0.49 | 1.1E-09 |
| 49081 | *egl6, cel74a* | GH74 | 1164 | 1716 | 0.56 | 2.5E-09 |
| 82227 | *cel3c* | GH3 | 76 | 133 | 0.82 | 4.5E-08 |
| 81598 | *chi18-7* | GH18 | 125 | 27 | -2.24 | 1.1E-07 |
| 73256 |  | GH81 | 0 | 9 | 5.18 | 3.6E-07 |
| 120229 | *xyn3* | GH10 | 7612 | 11118 | 0.55 | 4.9E-07 |
| 112140 |  | GH28 | 28 | 53 | 0.91 | 6.4E-07 |
| 60085 |  | GH31 | 3 | 12 | 1.81 | 2.9E-06 |
| 77299 |  | GH2 | 32 | 53 | 0.71 | 3.8E-06 |
| 3739 |  | GH43 | 136 | 171 | 0.33 | 5.0E-06 |
| 57128 |  | GH13 | 33 | 9 | -1.85 | 1.0E-05 |
| 123232 | *egl3* | GH12 | 16963 | 18535 | 0.13 | 1.7E-05 |
| 82616 | *egl8, cel5b* | GH5 | 61 | 81 | 0.41 | 2.5E-05 |
| 123368 |  | GH13 | 130 | 42 | -1.62 | 3.1E-05 |
| 59689 |  | GH2 | 8 | 1 | -3.80 | 4.2E-05 |
| 120312 | *egl2, cel5a* | GH5 | 11797 | 12657 | 0.10 | 4.2E-05 |
| 44214 | *axe2* | CE5 | 84 | 21 | -1.98 | 4.4E-05 |
| 77284 |  | GH12 | 86 | 129 | 0.59 | 5.3E-05 |
| 75036 |  | GH63 | 41 | 12 | -1.76 | 9.6E-05 |
| 67844 |  | GH76 | 89 | 156 | 0.81 | 1.1E-04 |
| 59082 | *chi18-1* | GH18 | 20 | 1 | -4.52 | 1.5E-04 |
| 104797 | *cel3f* | GH3 | 23 | 33 | 0.53 | 1.8E-04 |
| 122511 |  | GH16 | 110 | 33 | -1.75 | 2.3E-04 |
| 60635 |  | GH92 | 6 | 0 | -6.38 | 3.1E-04 |
| 79921 |  | GH92 | 11 | 2 | -2.82 | 4.7E-04 |
| 72488 |  | GH95 | 9 | 14 | 0.67 | 7.0E-04 |
| 72339 | *chi18-9* | GH18 | 3 | 7 | 1.16 | 8.4E-04 |
| 122081 | *egl1* | GH7 | 11506 | 11918 | 0.05 | 1.4E-03 |
| 124016 |  | GH36 | 18 | 4 | -2.05 | 2.1E-03 |
| 55802 |  | GH76 | 323 | 319 | -0.02 | 2.6E-03 |
| 65333 |  | GH15 | 30 | 8 | -1.82 | 3.1E-03 |
| 65380 |  | GH47 | 54 | 61 | 0.18 | 4.4E-03 |
