# Supplementary material for: Disruption of alpha-tubulin releases carbon catabolite repression and enhances enzyme production in Trichoderma reesei even in the presence of glucose
Source: Biotechnol Biofuels. 2021 Feb 8;14:39. doi: 10.1186/s13068-021-01887-0 (PMC7869464; doi:10.1186/s13068-021-01887-0)
Supplement: Supplementary file 5 — Additional file 5: Table S2. RPKMs of major CAZymes. [file 13068_2021_1887_MOESM5_ESM.docx]

# Table S6: Enriched gene ontology categories of DEGs between PC-3-7 and PC-3-7Δ*tubB*

|  | *p*-value | Number of genes | Number of DEGs | Number of upregulated DEGs in PC-3-7Δ*tubB* | GO term |
| --- | --- | --- | --- | --- | --- |
| GO:0005975 | 3.04E-17 | 141 | 56 | 43 | carbohydrate metabolic process |
| GO:0004553 | 4.65E-16 | 83 | 40 | 36 | hydrolase activity, hydrolyzing O-glycosyl compounds |
| GO:0006810 | 9.99E-12 | 350 | 88 | 79 | transport |
| GO:0016020 | 4.33E-10 | 379 | 88 | 76 | membrane |
| GO:0030248 | 2.01E-08 | 14 | 11 | 11 | cellulose binding |
| GO:0016021 | 3.25E-08 | 386 | 83 | 65 | integral component of membrane |
| GO:0005576 | 3.99E-08 | 35 | 17 | 15 | extracellular region |
| GO:0008152 | 6.69E-08 | 515 | 101 | 62 | metabolic process |
| GO:0006865 | 6.73E-08 | 44 | 20 | 19 | amino acid transport |
| GO:0016491 | 1.16E-06 | 452 | 88 | 45 | oxidoreductase activity |
| GO:0005351 | 4.21E-06 | 59 | 21 | 17 | carbohydrate:proton symporter activity |
| GO:0005215 | 5.97E-06 | 251 | 56 | 47 | transporter activity |
| GO:0008643 | 1.08E-05 | 53 | 19 | 16 | carbohydrate transport |
| GO:0008733 | 1.25E-05 | 49 | 18 | 15 | L-arabinose isomerase activity |
| GO:0006526 | 8.11E-05 | 8 | 6 | 5 | arginine biosynthetic process |
| GO:0004567 | 1.65E-04 | 6 | 5 | 4 | beta-mannosidase activity |
| GO:0006520 | 2.66E-04 | 28 | 11 | 9 | cellular amino acid metabolic process |
| GO:0003824 | 4.73E-04 | 402 | 71 | 43 | catalytic activity |
| GO:0008810 | 1.31E-03 | 3 | 3 | 3 | cellulase activity |
| GO:0004034 | 1.68E-03 | 3 | 3 | 1 | aldose 1-epimerase activity |
| GO:0004084 | 1.84E-03 | 3 | 3 | 3 | branched-chain-amino-acid transaminase activity |
| GO:0006012 | 2.59E-03 | 6 | 4 | 1 | galactose metabolic process |
| GO:0005375 | 2.73E-03 | 4 | 3 | 3 | copper ion transmembrane transporter activity |
| GO:0008652 | 2.89E-03 | 6 | 4 | 4 | cellular amino acid biosynthetic process |
| GO:0008422 | 2.98E-03 | 13 | 6 | 6 | beta-glucosidase activity |
| GO:0006465 | 3.09E-03 | 4 | 3 | 3 | signal peptide processing |
| GO:0006096 | 4.26E-03 | 14 | 6 | 2 | glycolytic process |
| GO:0004040 | 4.66E-03 | 14 | 6 | 2 | amidase activity |
| GO:0000105 | 4.80E-03 | 7 | 4 | 4 | histidine biosynthetic process |
| GO:0016597 | 5.31E-03 | 7 | 4 | 4 | amino acid binding |
| GO:0006807 | 6.65E-03 | 20 | 7 | 5 | nitrogen compound metabolic process |
