# Supplementary material for: Disruption of alpha-tubulin releases carbon catabolite repression and enhances enzyme production in Trichoderma reesei even in the presence of glucose
Source: Biotechnol Biofuels. 2021 Feb 8;14:39. doi: 10.1186/s13068-021-01887-0 (PMC7869464; doi:10.1186/s13068-021-01887-0)
Supplement: Supplementary file 6 — Additional file 6: Table S3. RPKM values of transcription factors. [file 13068_2021_1887_MOESM6_ESM.docx]

# Table S7: The differentially expressed predicted sugar transporter genes

| Gene ID | Gene name | RPKM | | Log_2_ Fold Change of RPMK | FDR |
| --- | --- | --- | --- | --- | --- |
|  |  | PC-3-7_48h_C | Δ*tubB* _48h_C |  |  |
| 69957 | *Tr69957* | 2 | 156 | 6.15 | 2.0E-43 |
| 46819 |  | 6 | 80 | 3.79 | 2.3E-39 |
| 56684 |  | 103 | 305 | 1.57 | 4.2E-32 |
| 50894 | *str1* | 818 | 1561 | 0.93 | 2.1E-21 |
| 5656 |  | 10 | 51 | 2.29 | 2.5E-18 |
| 3405 | *crt1* | 1980 | 4142 | 1.06 | 1.6E-15 |
| 50618 |  | 36 | 75 | 1.05 | 5.0E-12 |
| 48444 |  | 7 | 24 | 1.82 | 3.4E-10 |
| 121482 | *str2* | 2 | 129 | 6.11 | 5.8E-10 |
| 65191 |  | 1 | 17 | 4.85 | 1.7E-09 |
| 79202 | *TR79202* | 1217 | 1925 | 0.66 | 4.2E-09 |
| 106330 |  | 60 | 93 | 0.63 | 1.6E-08 |
| 77517 | *TR77517* | 77 | 135 | 0.80 | 3.5E-07 |
| 80091 |  | 3 | 14 | 2.04 | 7.8E-07 |
| 69901 |  | 0 | 14 | 5.24 | 7.9E-07 |
| 68812 |  | 26 | 3 | -3.24 | 1.1E-06 |
| 81670 |  | 92 | 114 | 0.31 | 1.5E-06 |
| 53903 |  | 1 | 9 | 3.35 | 4.6E-06 |
| 57088 |  | 20 | 31 | 0.65 | 1.1E-04 |
| 60945 |  | 109 | 35 | -1.64 | 1.8E-04 |
| 81659 |  | 14 | 0 | -6.42 | 2.6E-04 |
| 122013 |  | 171 | 61 | -1.48 | 3.3E-04 |
| 77785 |  | 61 | 72 | 0.23 | 1.9E-03 |
