# Supplementary material for: Disruption of alpha-tubulin releases carbon catabolite repression and enhances enzyme production in Trichoderma reesei even in the presence of glucose
Source: Biotechnol Biofuels. 2021 Feb 8;14:39. doi: 10.1186/s13068-021-01887-0 (PMC7869464; doi:10.1186/s13068-021-01887-0)
Supplement: Supplementary file 7 — Additional file 7: Table S4. Correlation matrix of RPKMs between all RNA-seq conditions. [file 13068_2021_1887_MOESM7_ESM.docx]

# Table S8: PCR primers

| Primer name | Primer sequence |
| --- | --- |
| swaI *tubB* F | 5' CTAGAGTATTTAAATTTTCGCCTTAGCGGTTCCAT 3' |
| swaI *tubB* R | 5' TGCAGGTATTTAAATTTGGTTCTTGGTTTGGAGGG 3' |
| swaI pUC F | 5' ATTTAAATACCTGCAGGCATGCAAGCTT 3' |
| swaI pUC R | 5' ATTTAAATACTCTAGAGGATCCCCGGGT 3' |
| *tubBback*-*amdS* F | 5' CGTTTCCAGTGCGCAAAGTACCGCGCGCTTGACAA 3' |
| *tubBfront*-*amdS* R | 5' CCAATGATGTGCGCATCTGGGAAATGTTCTTTGGC 3' |
| *amdS* F | 5' TGCGCACATCATTGGATAGG 3' |
| *amdS* R | 5' TGCGCACTGGAAACGCAACC 3' |
| *tubBfront-Pegl1* F | 5' AGAACATTTCCCAGATTTCAGCAATGCGTGGCGTT 3' |
| *tubBfront* R | 5' TCTGGGAAATGTTCTTTGGCAATA 3' |
| Primer1 | 5' TTAATACTCGGCAGTCTCGTCAG 3' |
| Primer2 | 5' TCAAAGACCTACTGAACCCG 3' |
| Primer3 | 5' ATGAGAGGCGAGGTCAGTGT 3' |
| Primer4 | 5' TTTCGCCTTAGCGGTTCCAT 3' |
