# Supplementary material for: Disruption of alpha-tubulin releases carbon catabolite repression and enhances enzyme production in Trichoderma reesei even in the presence of glucose
Source: Biotechnol Biofuels. 2021 Feb 8;14:39. doi: 10.1186/s13068-021-01887-0 (PMC7869464; doi:10.1186/s13068-021-01887-0)
Supplement: Supplementary file 8 — Additional file 8: Table S5. CAZymes that were significantly changed in the PC-3-7ΔtubB compared to PC-3-7. [file 13068_2021_1887_MOESM8_ESM.docx]

# Table S9: Plasmids, PCR templates and primers used for plasmid construction

| Plasmid | Template | Forward primer | Reverse primer |
| --- | --- | --- | --- |
| pUC-*tubB* | gDNA of PC-3-7 | swaI *tubB* F | swaI *tubB* R |
|  | pUC118 | swaI pUC F | swaI pUC R |
| pUC-Δ*tubB*-*amdS* | pUC-*tubB* | *tubBback*-*amdS* F | *tubBfront*-*amdS* R |
|  | pUC-*amdS* | *amdS* F | *amdS* R |
| pUC-Δ*tubB*-*Pegl1-aabgl1-amdS* | gDNA of E1AB1 | *tubBfront-Pegl1* F | *amdS* R |
|  | pUC-*tubB* | *tubBback*-*amdS* F | *tubBfront* R |
