# Supplementary material for: Disruption of alpha-tubulin releases carbon catabolite repression and enhances enzyme production in Trichoderma reesei even in the presence of glucose
Source: Biotechnol Biofuels. 2021 Feb 8;14:39. doi: 10.1186/s13068-021-01887-0 (PMC7869464; doi:10.1186/s13068-021-01887-0)
Supplement: Supplementary file 11 — Additional file 11: Table S8. PCR primers. [file 13068_2021_1887_MOESM11_ESM.docx]

# Table S1. RPKMs of tubulins

| Gene ID | Gene name | PC-3-7_  24h_C | Δ*tubB* _  24h_C | PC-3-7_  24h_C+G | Δ*tubB* 24h_C+G | PC-3-7_  48h_C | Δ*tubB* _  48h_C | PC-3-7_  48h_C+G | Δ*tubB* _  48h_C+G |
| --- | --- | --- | --- | --- | --- | --- | --- | --- | --- |
| 122886 | tubulin beta chain 2 | 230 | 241 | 260 | 345 | 260 | 176 | 440 | 216 |
| 120789 | tubulin alpha chain 2 | 194 | 168 | 180 | 299 | 260 | 169 | 409 | 181 |
| 21742 | tubulin beta chain 1 | 82 | 55 | 59 | 82 | 106 | 52 | 167 | 46 |
| 104790 | tubulin gamma chain | 8 | 7 | 11 | 13 | 8 | 6 | 16 | 7 |
| 120830 | tubulin alpha chain 1(*tubB*) | 49 | 0 | 60 | 0 | 79 | 0 | 119 | 0 |
