# Supplementary figures and images for: Disruption of alpha-tubulin releases carbon catabolite repression and enhances enzyme production in Trichoderma reesei even in the presence of glucose
Source: Biotechnol Biofuels. 2021 Feb 8;14:39. doi: 10.1186/s13068-021-01887-0 (PMC7869464; doi:10.1186/s13068-021-01887-0)

Figure S2 N. Shibata *et. al.*

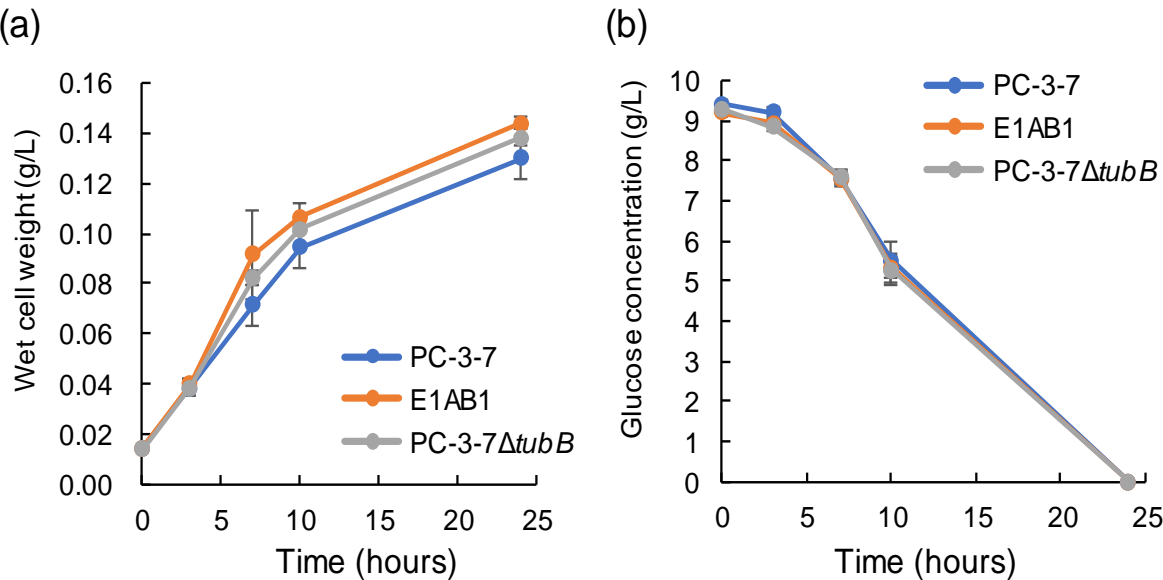

Supplement: Supplementary file 12 — Additional file 12: Table S9. Plasmids, PCR templates and primers used for plasmid construction. [file 13068_2021_1887_MOESM12_ESM.pdf]

Figure S3 N. Shibata *et. al.*

(a)

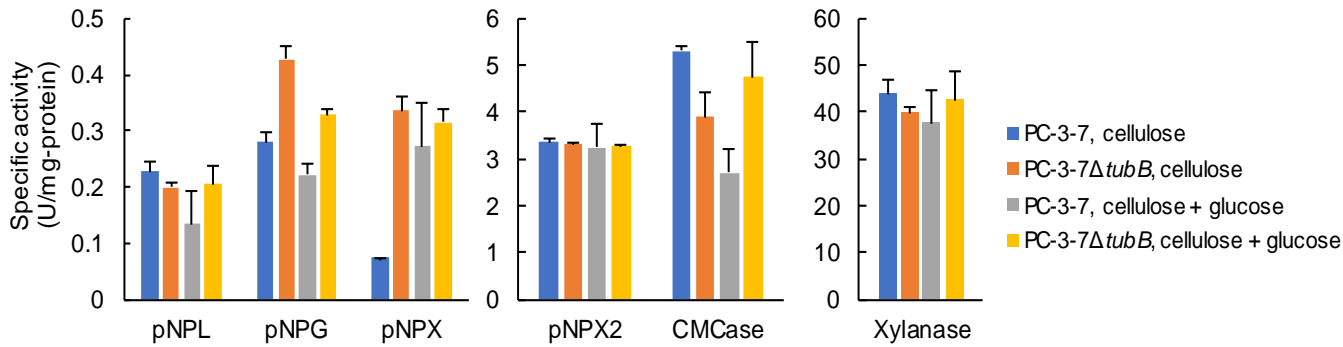

(b)

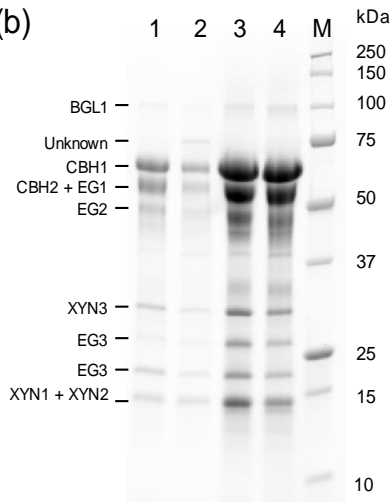

(c)

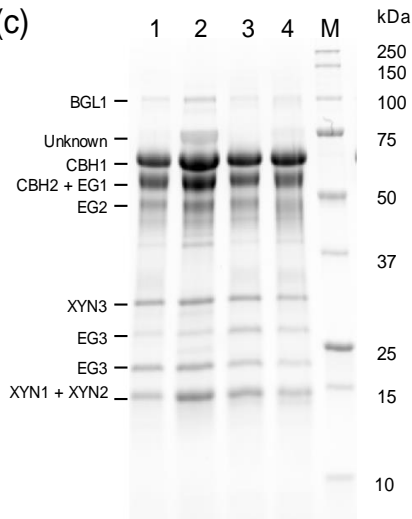

Supplement: Supplementary file 13 — Additional file 13: Table S10. Matrix table with raw read counts. [file 13068_2021_1887_MOESM13_ESM.pdf]

Figure S1 N. Shibata *et. al.*

(a)

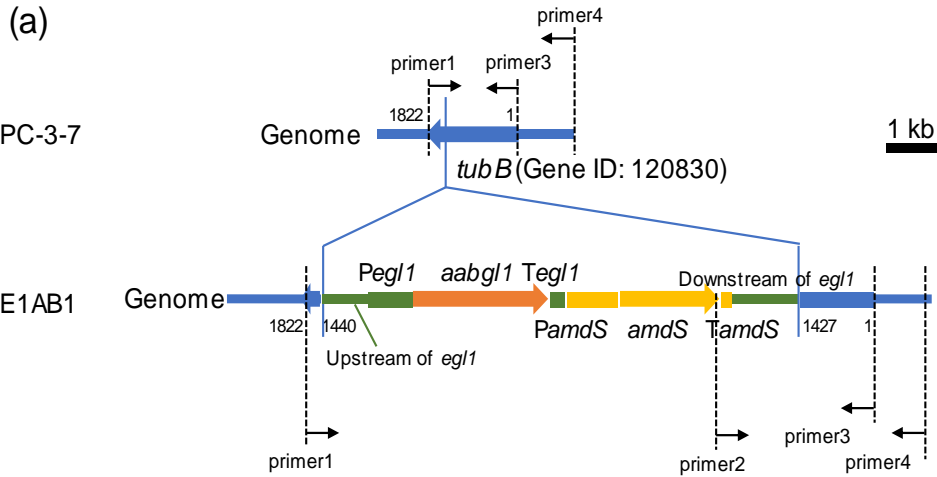

(b)

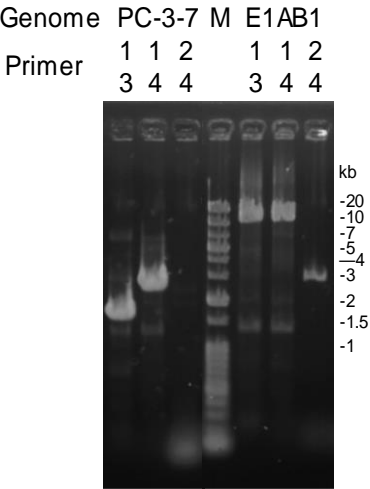

Supplement: Supplementary file 14 — Additional file 14: Table S11. RPKM values for every gene. [file 13068_2021_1887_MOESM14_ESM.pdf]
